# A network pharmacology and molecular docking approach to reveal the mechanism of Chaihu Anxin Capsule in depression

**DOI:** 10.3389/fendo.2023.1256045

**Published:** 2023-09-07

**Authors:** Lin Yang, Yan Zhao, Ruochen Qu, Yan Fu, Chunhua Zhou, Jing Yu

**Affiliations:** ^1^ Department of Pharmacy, First Hospital of Hebei Medical University, Shijiazhuang, China; ^2^ Core Facilities and Centers, Hebei Medical University, Shijiazhuang, China; ^3^ School of Pharmaceutical Sciences, Hebei Medical University, Shijiazhuang, China

**Keywords:** traditional chinese medicine, network pharmacology, molecular docking, depression, Chaihu Anxin capsule

## Abstract

**Introduction:**

As one of the most frequently diagnosed mental disorders, depression is expected to become the most common disease worldwide by 2030. Previous studies have shown that Chaihu Anxin Capsule has powerful antidepressant effects. However, its mechanisms are not fully understood. The aim of our research is to reveal the mechanisms of Chaihu Anxin Capsule in treating depression.

**Methods:**

Information about the ingredients of the herb was gathered using the TCMSP. Genes associated with antidepressants were gathered from the GeneCards database. An “herbal-ingredient-target” network was constructed and analyzed using Cytoscape software. The PPI network of the antidepressant targets of Chaihu Anxin Capsule was constructed using the STRING database. KEGG pathway and GO enrichment were used to analyze the antidepressant targets. Molecular docking technology was used to confirm the capacity of the primary active ingredients of Chaihu Anxin Capsule to bind to central targets using AutoDock Vina and PyMOL software.

**Results:**

Network analysis showed that five targets might be therapeutic targets of Chaihu Anxin Capsule in depression, namely, JUN, IL6, AKT1, TP53, and STAT3. The gene enrichment analysis implied that Chaihu Anxin Capsule benefits patients with depression by modulating pathways related to lipids and atherosclerosis and the AGE-RAGE signaling pathway in diabetic complications. Molecular docking analyses revealed that JUN, IL6, AKT1, TP53, and STAT3 had good affinities for quercetin, beta-sitosterol and kaempferol.

**Conclusion:**

According to the bioinformatics data, the antidepressant effects of Chaihu Anxin Capsule may be primarily linked to cholesterol and atherosclerosis as well as the AGE-RAGE signaling pathway in diabetic complications. These results emphasize that the expected therapeutic targets may be possible indicators for antidepressant activity.

## 1 Introduction

Depression, as a severe affective mental disorder, is accompanied by a loss of enjoyment and impairment of cognition, behavior, and autonomic nerve function, which seriously affect the social function of patients (1). The frequency of depression has significantly increased because of significant changes in physical, psychological, and social interactions; in the United States, severe depression affects 21% of women and 11%–13% of men over the course of their lifespan (2). Depression is expected to become the most common disease worldwide by 2030 (3). Therefore, research on the pathogenesis, prevention, and treatment of depression has become a current hotspot.

Traditional Chinese medicine (TCM) has a clinical application history of thousands of years, playing a role through multiple links, targets, and steps. TCM is widely accepted because it comes from nature and has mild effects and few side effects. Therefore, studying the efficiency and mechanism of TCM in preventing and curing depression has attracted increasing attention from experts worldwide (4). For example, Jieyu Pills, Jiawei Xiaoyao capsule, Shugan Jieyu capsule, and others have proven high clinical effectiveness when used to prevent and treat depression (5–7).

Chaihu Anxin Capsule, the Hebei Medical University first hospital in-hospital preparation, is composed of *Bupleuri radix*, *Paeoniae Radix Alba*, *Puerariae Radix* and other medicinal materials and is used to treat depression syndrome with good clinical effects. Wang G et al. identified the antidepressant effect of *Puerariae Radix* with the help of network pharmacology (8). It has also been established that Radix Bupleuri and Radix Paeoniae Alba have an impact on depression (9). Previous studies have demonstrated that Chaihu Anxin capsules can significantly improve depression-like behavior in rats induced by reserpine and reduce corticosterone levels and hippocampal neuron damage (10). However, because of the complex composition of the Chaihu Anxin capsule, its primary material basis and molecular mechanism for depression therapy remain unknown.

The current study sought to systematically investigate the anticipated therapeutic targets and biological signaling pathways of Chaihu Anxin Capsule against depression based on network pharmacology and molecular docking and to further provide bioinformatics data for follow-up clinical and basic research on the treatment of Chaihu Anxin Capsule.

## 2 Materials and methods

### 2.1 Screening of active ingredients and target genes

Oral bioavailability (OB) is the rate at which a medicine taken orally and absorbed in the gastrointestinal system enters the bloodstream through the liver. Drug-likeness (DL) is the degree to which herbal components resemble recognized drugs structurally. With the help of the TraditionalChineseMedicineSystemsPharmacology Database and Analysis Platform (TCMSP, https://old.tcmsp-e.com/tcmsp.php/) (11), the chemical constituents in *Paeoniae Radix Alba*, *Radix Bupleuri*, *Arum Ternatum Thunb*, *Jujubae Fructus*, *Poria Cocos*, *licorice*, *Radix Puerariae*, *Cinnamomi Ramulus*, *Zingiber Officinale Roscoe*, *Prunellae Spica* and *Corydalis Rhizoma* in Chaihu Anxin Capsule with the standard of OB ≥30% and DL ≥0.18 were retrieved as the active ingredients in this study (12). Moreover, text mining was used to incorporate compounds that did not fulfil the screening requirements but were described as metabolic regulators (13, 14).

### 2.2 Construction of an active ingredient-target network of the Chaihu Anxin capsule

The target proteins of the Chaihu Anxin capsule were identified using the UniProt database (https://www.uniprot.org/), and the targets underwent a unified conversion to abbreviated gene names (15). The Chaihu Anxin capsule’s active components and the relevant targets were imported into Cytoscape 3.9.1 to organize into a network (16).

### 2.3 Screening of disease targets

Targets connected to depression were found using the GeneCards (https://www.genecards.org/), OMIM (https://omim.org/), DrugBank (https://www.drugbank.ca/) and Therapeutic Target Database (https://db.idrblab.net/ttd/) databases in this study (17–20).

### 2.4 Screening of depression-related genes acted upon by the active ingredients of Chaihu Anxin Capsule

A Venn diagram was made to visualize the overlap between the genes of the active ingredients in Chaihu Anxin capsule and depression-related genes.

### 2.5 Construction of the herbal-active ingredient-antidepressant target network

To construct the “herbal-active ingredient-antidepressant target network” of Chaihu Anxin capsule, the intersecting genes and their active ingredients were imported into Cytoscape 3.9.1. The topological properties were analyzed with the “network analyzer” function.

### 2.6 Construction of a protein−protein interaction network and screening of its core targets

To obtain the relationships of PPls, the obtained intersecting genes were uploaded onto the STRING 11.5 platform (https://string-db.org/) (21). Apart from changing the minimal interaction threshold to “medium confidence” 0.7 with medium confidence and the species (protein species) to “Homo sapiens” (human), all other parameters were left at their default values. The top 30 intersection targets of Chaihu Anxin capsule against depression were identified.

### 2.7 GO and KEGG enrichment analyses

To explore the functions of the intersecting genes screened from the Chaihu Anxin capsule, we analyzed the intersecting genes as well as their roles in signaling pathways. The Kyoto Encyclopedia of Genes and Genomes (KEGG) and a functional enrichment and labelling tool based on Gene Ontology (GO) were both used to accomplish this. The Metascape database (https://metascape.org/gp/index.html#/main/step1) (22) was used as the source of the data.

### 2.8 Molecular docking

For the following molecular docking study, the most important gene among the core genes was chosen. The UniProt library was scanned for the receptor protein that the chosen gene codes for. A copy of the protein’s structure was made from the Research Collaboratory for Structural Bioinformatics Protein Data Bank (RCSB PDB) library (https://www.rcsb.org/) (23). The PubChem library (https://pubchem.ncbi.nlm.nih.gov) was used to obtain the structures of the ligand molecules (24). The receptor protein was dehydrated using PyMOL 2.5.0 software, and protein hydrogenations and charges were calculated using AutoDock 1.5.6 (25, 26). Active pocket sites where small molecule ligands attach were specified as parameters of the receptor protein docking site. The receptor protein was then docked with the small-molecule ligands of the Chaihu Anxin capsule’s active components using AutoDock Vina.

## 3 Results

### 3.1 Active ingredients and potential targets

The TCMSP was used to obtain information about the 260 active components in Chaihu Anxin capsule and their targets. OB≥30% and DL≥0.18 served as the criteria. There are 14 ingredients from *Paeoniae Radix Alba*, 19 ingredients from *Radix Bupleuri* and 6 ingredients from *Radix Puerariae*. These results are shown in **Table 1** (all ingredients are listed in **Supplementary Table 1**).

**Table 1:** Active ingredients of Paeoniae Radix Alba, Radix Bupleuri and Radix Puerariae.

Herbal	Mol ID	Molecule name	OB	DL
*Paeoniae Radix Alba*	MOL001921	Lactiflorin	49.12	0.80
*Paeoniae Radix Alba*	MOL001924	paeoniflorin	53.87	0.79
*Paeoniae Radix Alba*	MOL000211	Mairin	55.38	0.78
*Paeoniae Radix Alba*	MOL000358	beta-sitosterol	36.91	0.75
*Paeoniae Radix Alba*	MOL000359	sitosterol	36.91	0.75
*Paeoniae Radix Alba*	MOL001930	benzoyl paeoniflorin	31.27	0.75
*Paeoniae Radix Alba*	MOL001919	(3S,5R,8R,9R,10S,14S)-3,17-dihydroxy-4,4,8,10,14-pentamethyl-2,3,5,6,7,9-hexahydro-1H-cyclopenta[a]phenanthrene-15,16-dione	43.56	0.53
*Paeoniae Radix Alba*	MOL001925	paeoniflorin_qt	68.18	0.40
*Paeoniae Radix Alba*	MOL001910	11alpha,12alpha-epoxy-3beta-23-dihydroxy-30-norolean-20-en-28,12beta-olide	64.77	0.38
*Paeoniae Radix Alba*	MOL001918	paeoniflorgenone	87.59	0.37
*Paeoniae Radix Alba*	MOL001928	albiflorin_qt	66.64	0.33
*Paeoniae Radix Alba*	MOL000492	(+)-catechin	54.83	0.24
*Paeoniae Radix Alba*	MOL000422	kaempferol	41.88	0.24
*Paeoniae Radix Alba*	MOL000513	3,4,5-trihydroxybenzoic acid	31.69	0.04
*Radix Bupleuri*	MOL000449	Stigmasterol	43.83	0.76
*Radix Bupleuri*	MOL004718	伪-spinasterol	42.98	0.76
*Radix Bupleuri*	MOL002776	Baicalin	40.12	0.75
*Radix Bupleuri*	MOL004653	(+)-Anomalin	46.06	0.66
*Radix Bupleuri*	MOL013187	Cubebin	57.13	0.64
*Radix Bupleuri*	MOL004702	saikosaponin c_qt	30.50	0.63
*Radix Bupleuri*	MOL004598	3,5,6,7-tetramethoxy-2-(3,4,5-trimethoxyphenyl)chromone	31.97	0.59
*Radix Bupleuri*	MOL004624	Longikaurin A	47.72	0.53
*Radix Bupleuri*	MOL004609	Areapillin	48.96	0.41
*Radix Bupleuri*	MOL000354	isorhamnetin	49.60	0.31
*Radix Bupleuri*	MOL000490	petunidin	30.05	0.31
*Radix Bupleuri*	MOL004628	Octalupine	47.82	0.28
*Radix Bupleuri*	MOL000098	quercetin	46.43	0.28
*Radix Bupleuri*	MOL004648	Troxerutin	31.60	0.28
*Radix Bupleuri*	MOL000422	kaempferol	41.88	0.24
*Radix Bupleuri*	MOL004644	Sainfuran	79.91	0.23
*Radix Bupleuri*	MOL001645	Linoleyl acetate	42.10	0.20
*Radix Bupleuri*	MOL004637	Saikosaponin D	34.39	0.09
*Radix Bupleuri*	MOL004635	saikosaponin a	32.39	0.09
*Radix Puerariae*	MOL000358	beta-sitosterol	36.91	0.75
*Radix Puerariae*	MOL012297	puerarin	24.03	0.69
*Radix Puerariae*	MOL003629	Daidzein-4,7-diglucoside	47.27	0.67
*Radix Puerariae*	MOL002959	3’-Methoxydaidzein	48.57	0.24
*Radix Puerariae*	MOL000392	formononetin	69.67	0.21
*Radix Puerariae*	MOL000390	daidzein	19.44	0.19

### 3.2 Active ingredient-target network

**Figure 1:**
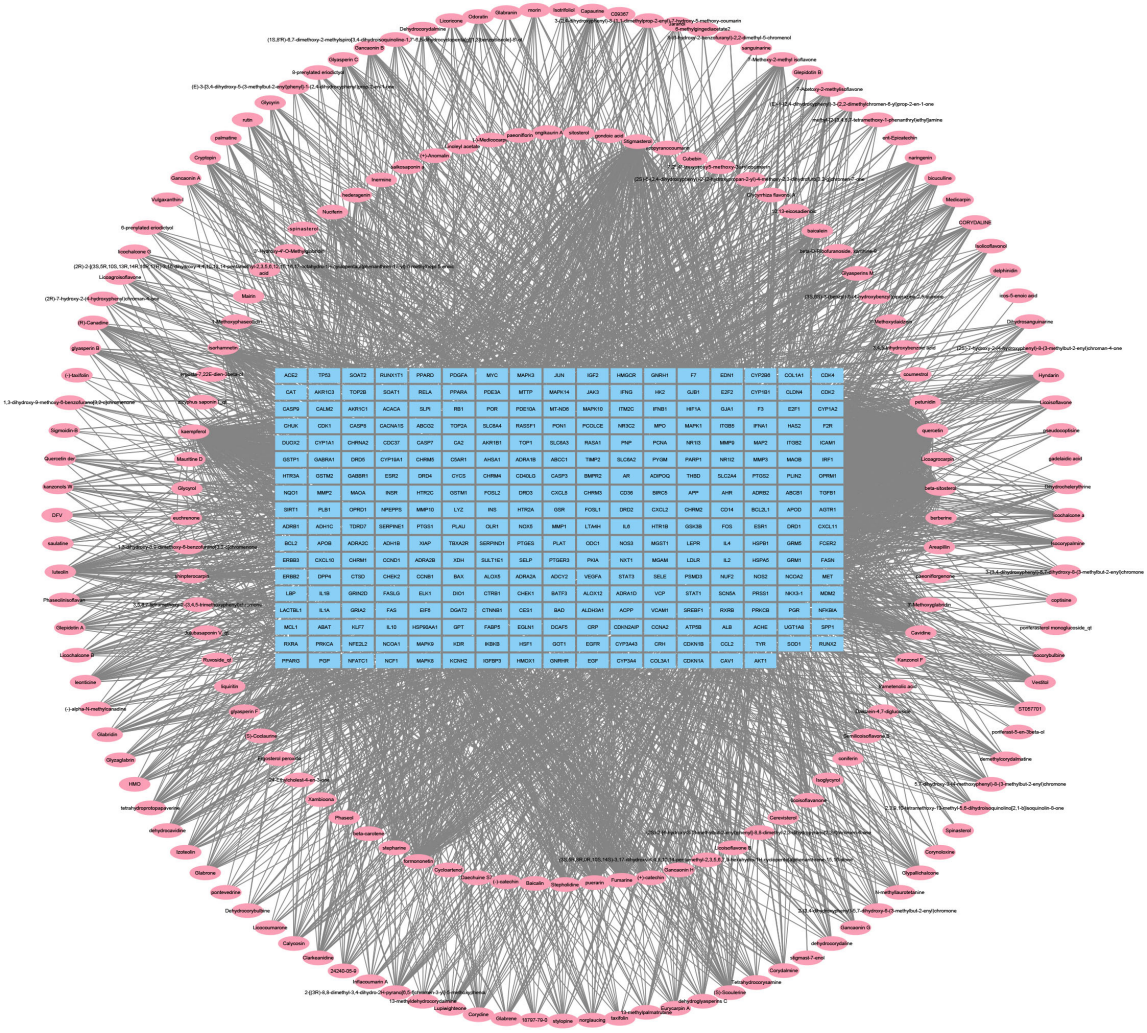
“Active ingredient-target” network construction. The blue rectangle nodes represent the targets; the pink elliptical nodes represent ingredients of Chaihu Anxin capsule.

The obtained ingredients in Chaihu Anxin Capsule and their potential targets were imported into Cytoscape software to construct the “active ingredient-target” network. There were 497 nodes and 4317 edges in the network. These results are shown in **Figure 1**. The blue rectangle nodes represent the targets; the pink elliptical nodes represent ingredients of Chaihu Anxin capsule.

### 3.3 Core action genes in the Chaihu Anxin capsule

**Figure 2:**
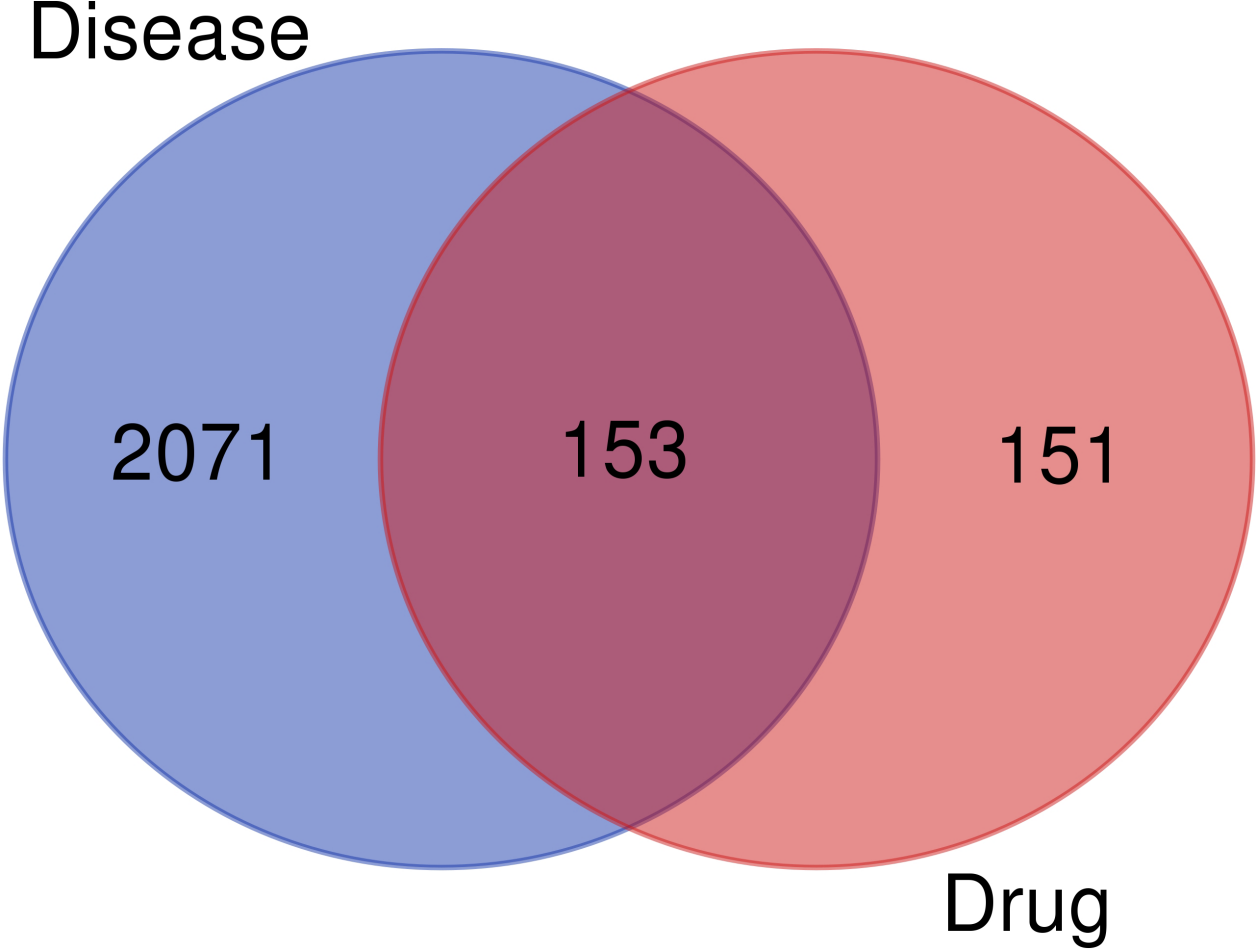
Venn diagram of the targets of Chaihu Anxin capsule and depression.

Using the term “antidepressant,” genes associated with depression were found from the GeneCards, Online Mendelian Inheritance in Man (OMIM), DrugBank and Therapeutic Target Database databases. Based on the junction results from the TCMSP and disease gene databases, gene intersections were created, and 153 intersection targets were acquired. These results are shown in **Figure 2** and **Supplementary Table 2**.

### 3.4 Herbal-active ingredients-antidepressant targets network

**Figure 3:**
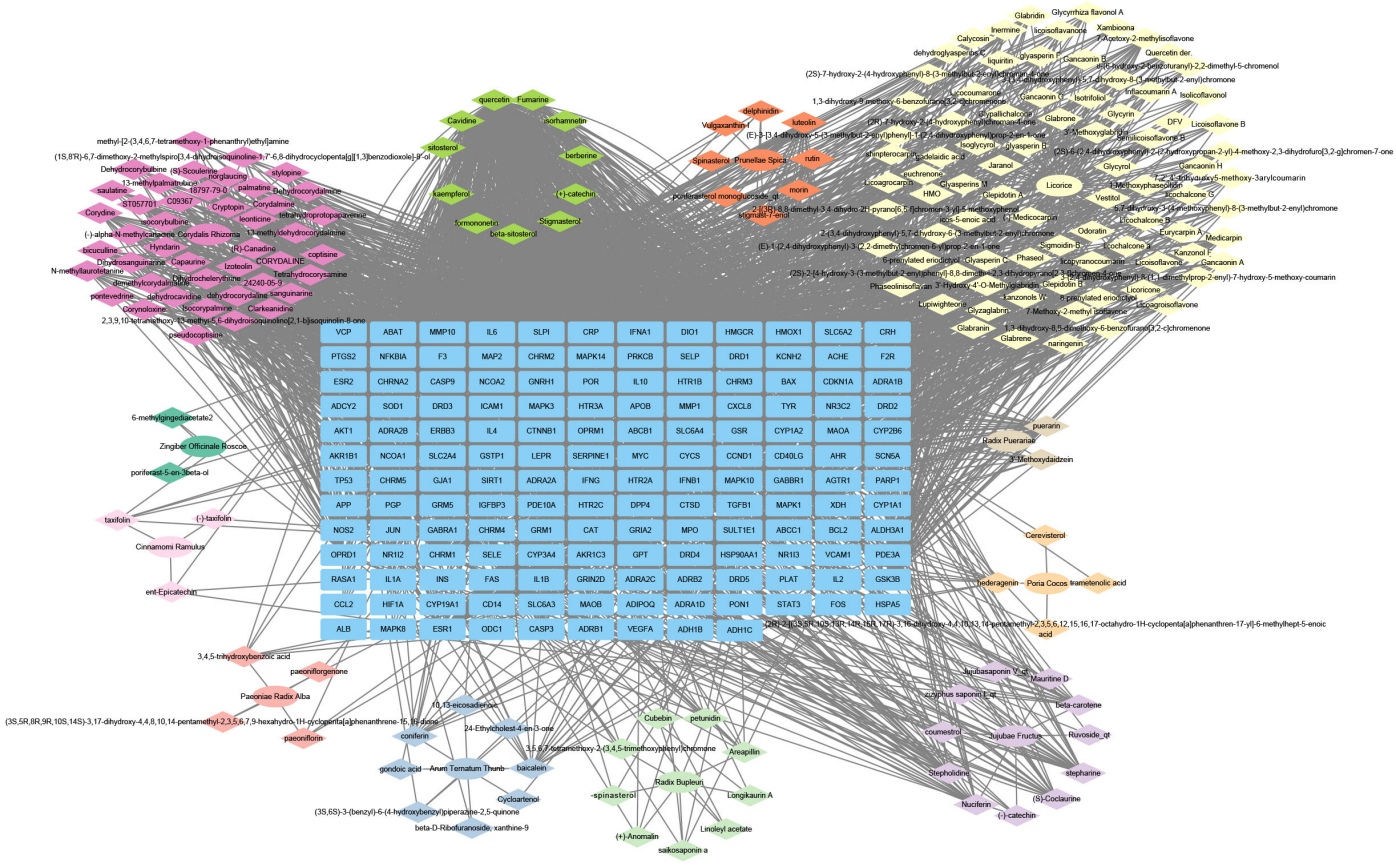
“Herbal-active ingredients-antidepressant target” network construction. The blue rectangle nodes represent the targets; the elliptical nodes represent herbals of Chaihu Anxin capsule; the diamond nodes represent ingredients of Chaihu Anxin capsule.

The intersection targets and their active ingredients in Chaihu Anxin Capsule were imported into Cytoscape 3.9.1 to structure the “herbal-active ingredients-antidepressant targets” network. There were 351 nodes and 2803 edges in the network. These results are shown in **Figure 3**. The blue rectangle nodes represent the targets; the elliptical nodes represent herbals of Chaihu Anxin capsule; the diamond nodes represent ingredients of Chaihu Anxin capsule. The active ingredients quercetin, beta-sitosterol, and kaempferol are the three ingredients with the highest “degree” values. These results are shown in **Table 2**.

**Table 2:** The three active ingredients with the highest degree value of Chaihu Anxin capsule.

Active ingredients	Degree
quercetin	350
beta-sitosterol	189
kaempferol	140

### 3.5 Construction and screening of a PPI between Chaihu Anxin capsule and depression

**Figure 4:**
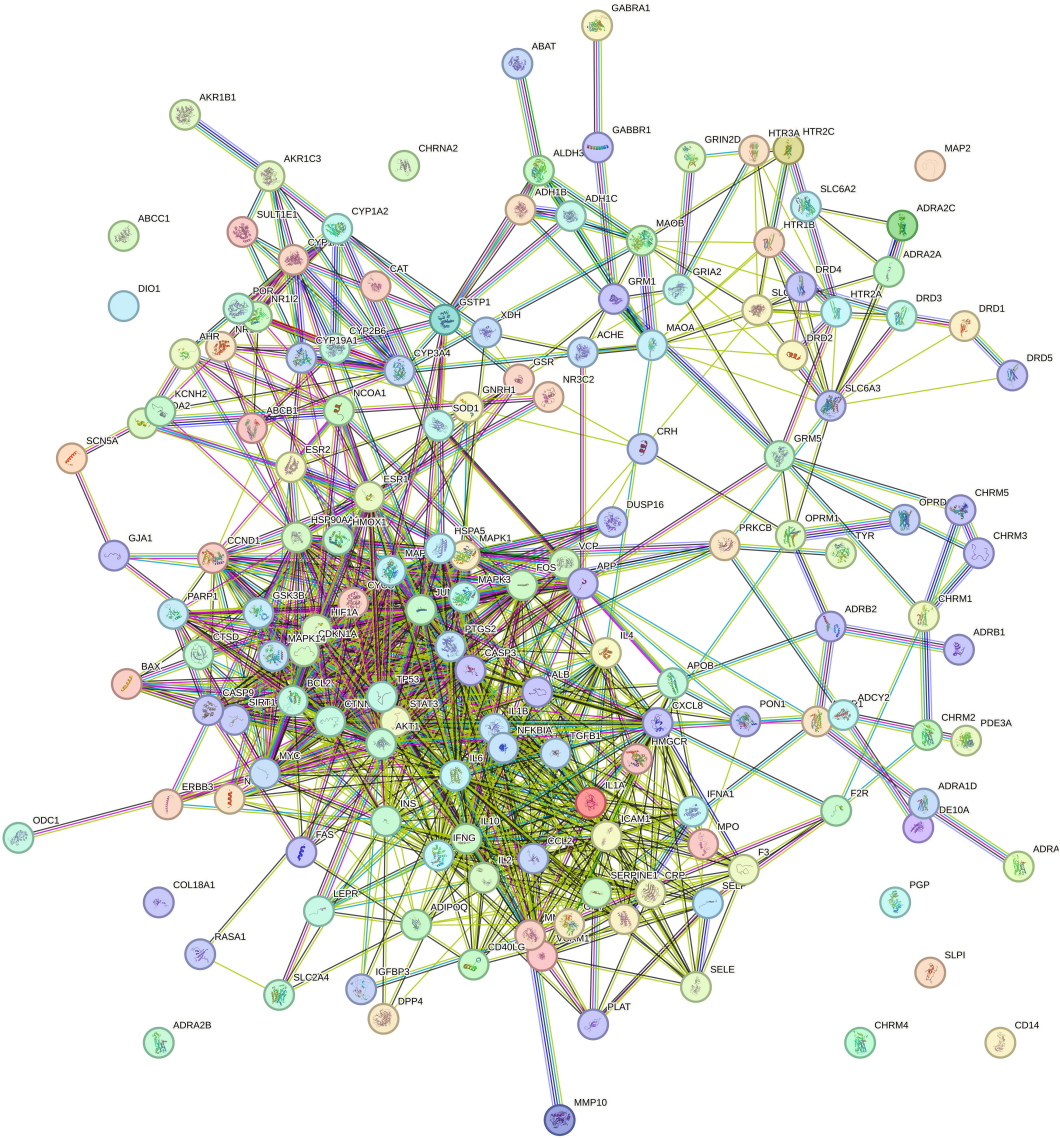
PPI network of the gene targets.

**Figure 5:**
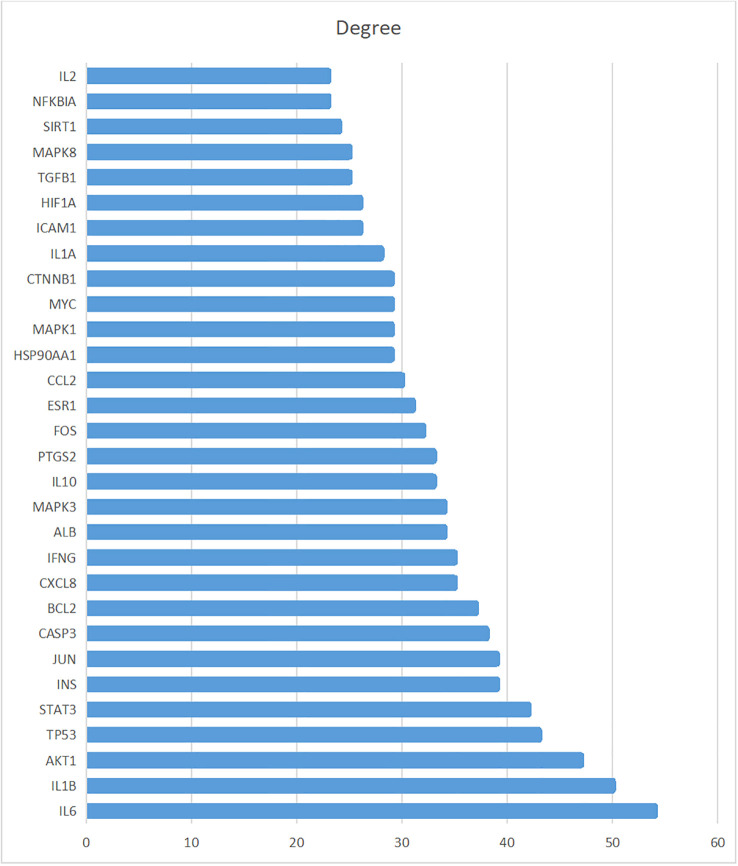
Thirty core targets determined in the PPI network.

The STRING database was used to investigate the 153 intersected genes and construct a PPI network. These results are shown in **Figure 4**. Targets with scores in the top 30 were chosen as core targets such as Interleukin-6 (IL6), Interleukin-1 beta (IL1B), RAC-alpha serine/threonine-protein kinase (AKT1), Cellular tumor antigen p53 (TP53), and Signal transducer and activator of transcription 3 (STAT3). These results are shown in **Figure 5** and **Table 3**.

**Table 3:** Thirty core targets.

Gene	Target name
IL6	Interleukin-6
IL1B	Interleukin-1 beta
AKT1	RAC-alpha serine/threonine-protein kinase
TP53	Cellular tumor antigen p53
STAT3	Signal transducer and activator of transcription 3
INS	Insulin
JUN	Transcription factor AP-1
CASP3	Caspase-3
BCL2	Apoptosis regulator Bcl-2
CXCL8	Interleukin-8
IFNG	Interferon gamma
ALB	Serum albumin
MAPK3	Mitogen-activated protein kinase 3
IL10	Interleukin-10
PTGS2	Prostaglandin G/H synthase 2
FOS	Proto-oncogene c-Fos
ESR1	Estrogen receptor
CCL2	C-C motif chemokine 2
HSP90AA1	Heat shock protein HSP 90
MAPK1	Mitogen-activated protein kinase 1
MYC	Myc proto-oncogene protein
CTNNB1	Catenin beta-1
IL1A	Interleukin-1 alpha
ICAM1	Intercellular adhesion molecule 1
HIF1A	Hypoxia-inducible factor 1-alpha
TGFB1	Transforming growth factor beta-1
MAPK8	Mitogen-activated protein kinase 8
SIRT1	NAD-dependent deacetylase sirtuin-1
NFKBIA	NF-kappa-B inhibitor alpha
IL2	Interleukin-2

### 3.6 KEGG pathway and GO enrichment analyses

**Figure 6:**
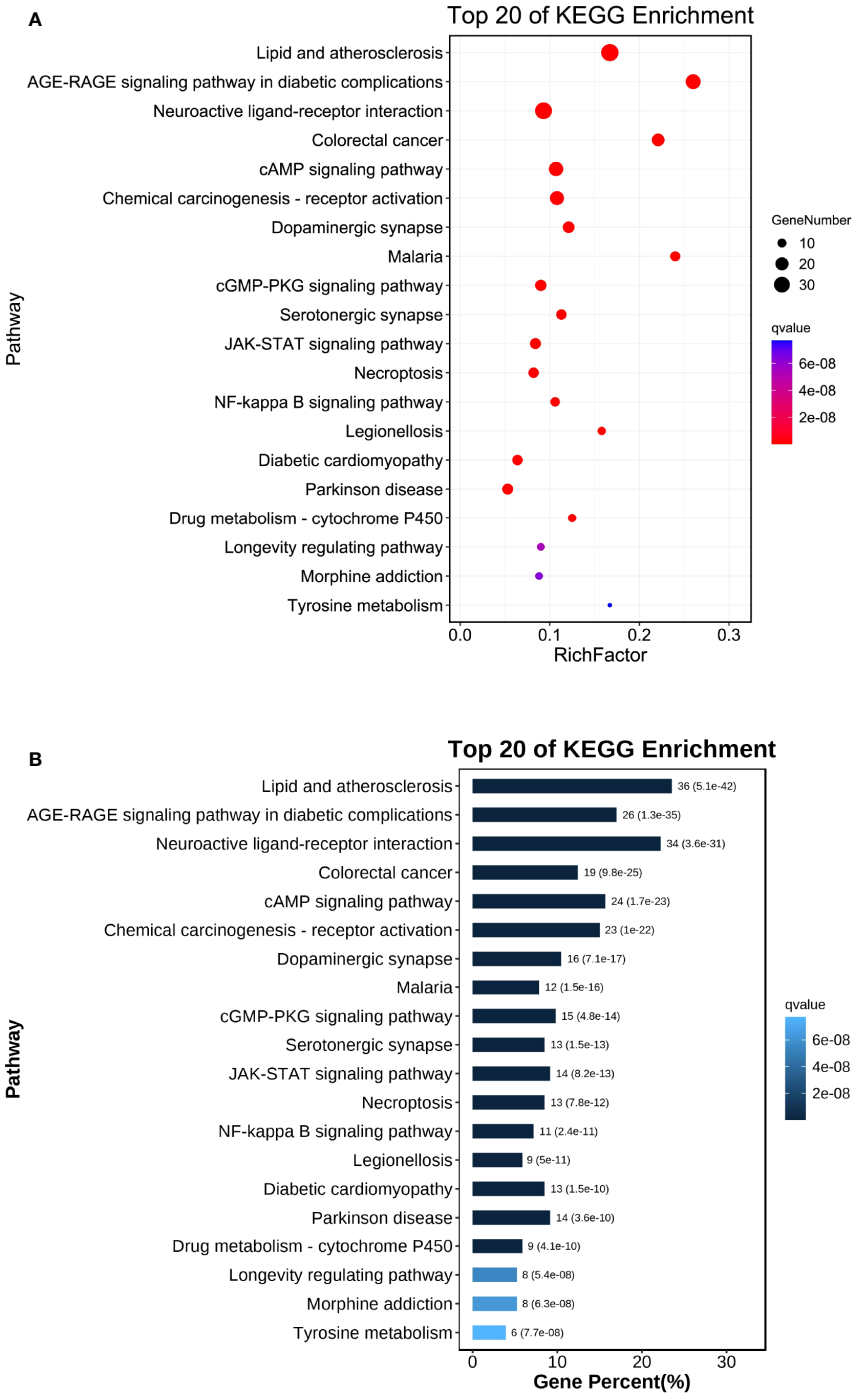
**(A)** Dot plot of KEGG enrichment analysis. **(B)** Bar plot of KEGG enrichment analysis. The size of the bubble represents the number of targets enriched in the indicated pathway, and the color of the bubble represents the p value of enrichment.

The 20 most enriched signaling pathways of Chaihu Anxin capsule were enriched and screened using KEGG pathways (P<0.05), including lipid and atherosclerosis and the AGE-RAGE signaling pathway in diabetic complications. These results are shown in **Figure 6**, **7**. The size of the bubble represents the number of targets enriched in the indicated pathway, and the color of the bubble represents the p value of enrichment. Nodes in red represent Chaihu Anxin Capsule-depression-related targets.

**Figure 7:**
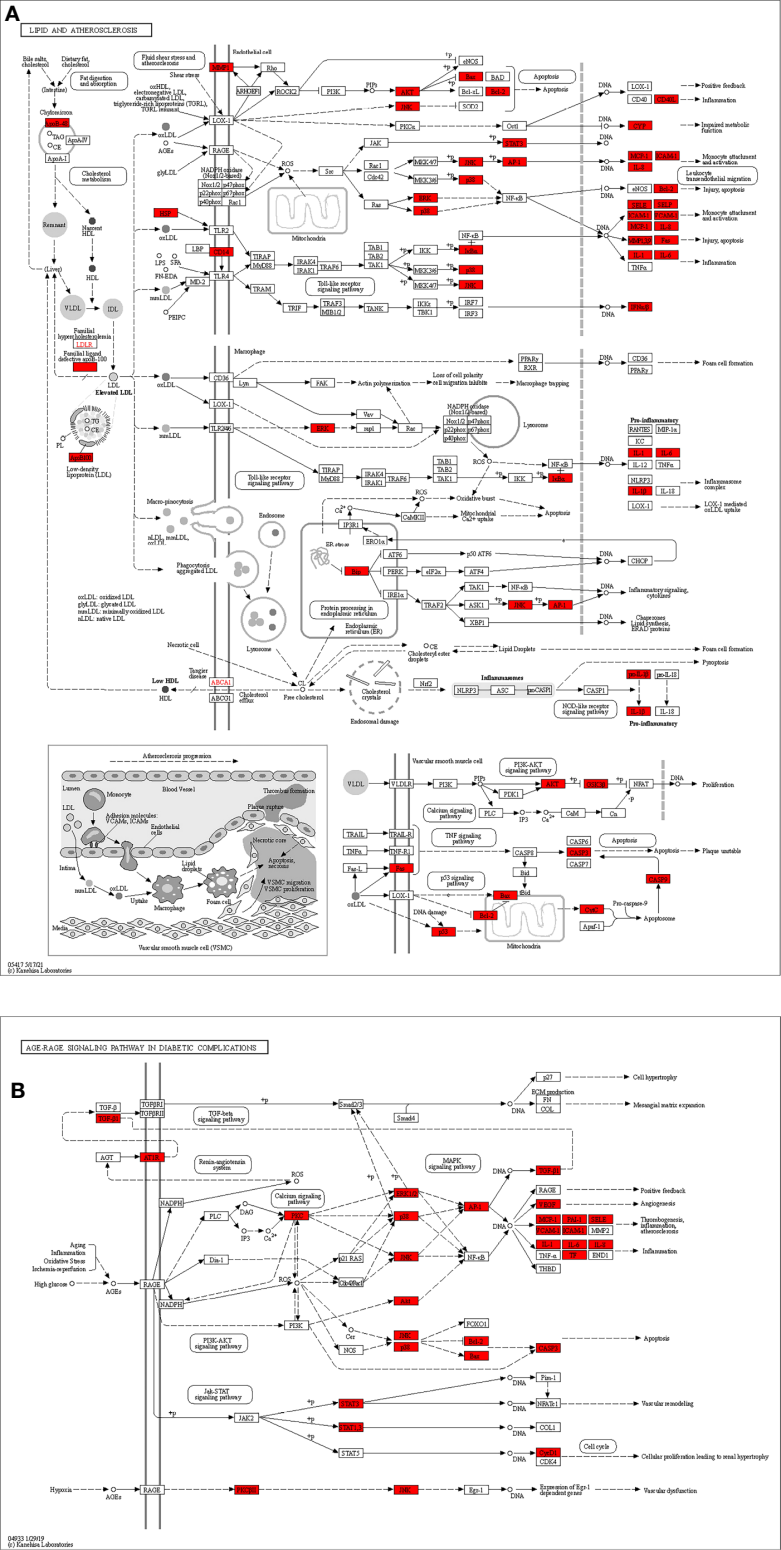
**(A)** Lipid and atherosclerosis and **(B)** AGE-RAGE signaling pathway in diabetic complications map of Chaihu Anxin Capsule for their potential treatment of depression. Nodes in red represent Chaihu Anxin Capsule-depression-related targets.

**Figure 8:**
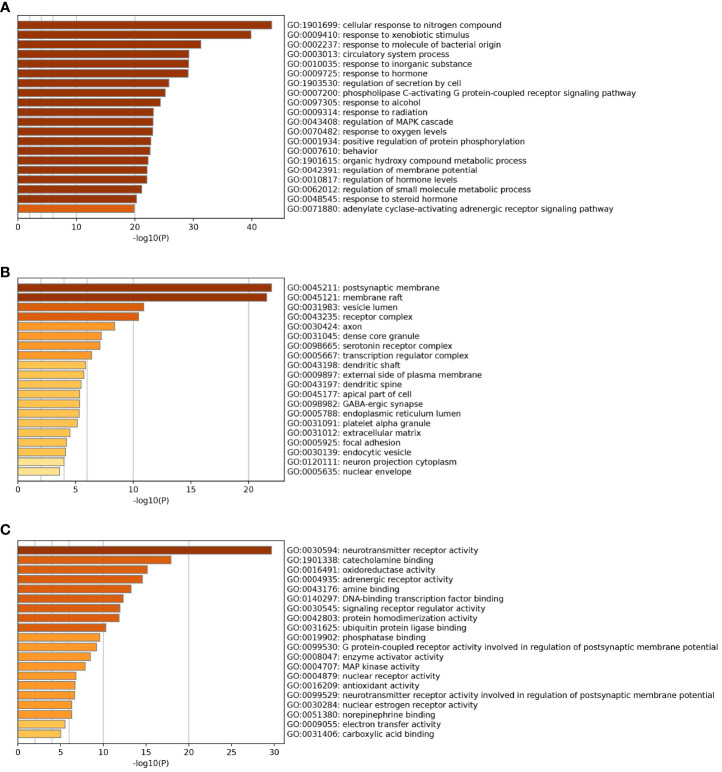
GO enrichment analysis. **(A)** Biological Processes. **(B)** Cellular Components. **(C)** Molecular Functions.

The 20 pathways with the highest GO enrichment of Chaihu Anxin capsule in biological processes, cellular components and molecular functions were identified by GO-based functional enrichment and annotation. These results are shown in **Figures 8A–C**. These targets pertain to cellular reactions to nitrogen compounds, xenobiotic stimuli, molecule of bacterial origin, neurotransmitter receptor activity, and postsynaptic membrane, among other things.

### 3.7 Molecular docking

**Figure 9:**
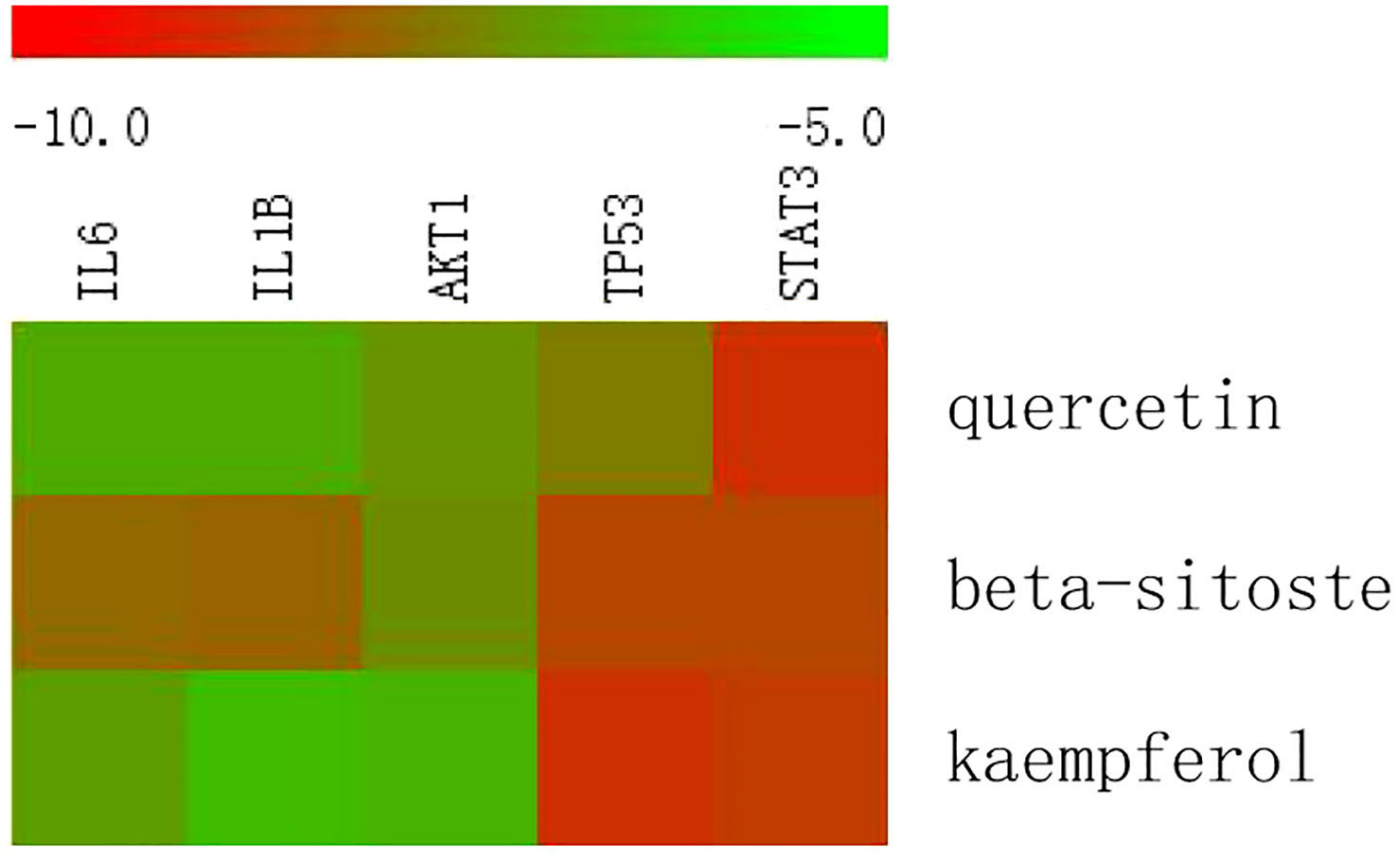
Molecular docking scores. (kcal·mol^-1^).

**Figure 10:**
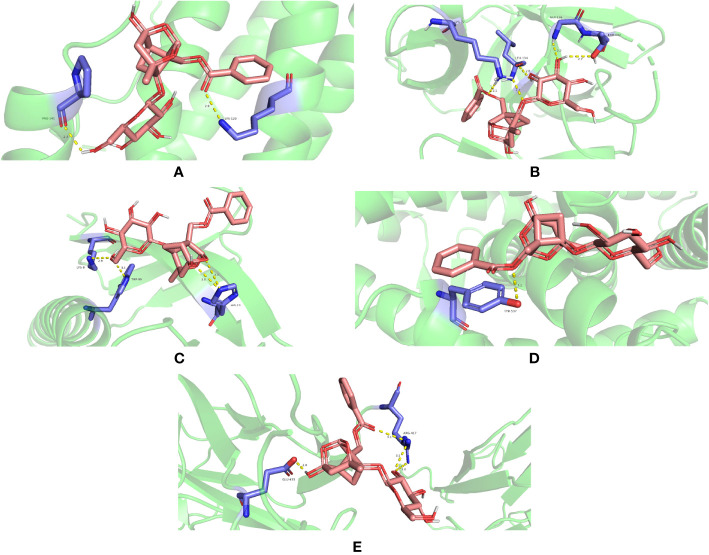
Molecular docking between puerarin and proteins **(A)** 1ALU, **(B)** 6Y8M, **(C)** 1UNP, **(D)** 1QKT, and **(E)** 6QHD (encoded by IL6, IL1B, AKT1, TP53, and STAT3, respectively).

IL6 (PDB ID 1ALU), IL1B (PDB ID 6Y8M), AKT1 (PDB ID 1UNP), TP53 (PDB ID 1QKT), and STAT3 (PDB ID 6QHD) were selected for molecular docking analysis. For molecular docking, the top three active substances (quercetin, beta-sitosterol, and kaempferol) in the network were chosen. Molecular docking results indicated that the binding energies of the three active ingredients with IL6, IL1B, AKT1, TP53, and STAT3 were less than -5.0 kcal·mol^-1^, showing good binding force. These results are shown in **Figure 9**. Through hydrogen bonding, quercetin binds to the amino acid residues of IL6 such as PRO-141 and LYS-120; binds to the amino acid residues of IL1B such as GLY-136, THR-137, LEU-134, and LYS-77; binds to the amino acid residues of AKT1 such as LYS-8, TRP-99 and HIS-13; binds to the amino acid residues of TP53 such as TYR-537; binds to the amino acid residues of STAT3 such as GLU-435 and ARG-417. The results of molecular docking of quercetin with five significant genes are presented in **Figures 10A–E** (all results of molecular docking are presented in **Supplementary Figure 1**).

## 4 Discussion

TCM has a full theoretical framework and a clinical application history of thousands of years with rich resources. TCM includes natural medicines with obvious curative effects, high efficiency, safety, and low toxicity and side effects for antidepressant treatment (4), which play a role through multiple links, targets, and steps. The Chaihu Anxin capsule is an in-hospital preparation from the First Hospital of Hebei Medical University and is composed of *Bupleuri radix*, *Paeoniae Radix Alba*, *Puerariae radix* and other medicinal materials. This medicine is used to treat depression syndrome, the symptoms of which include dizziness, asthenia, restlessness, dreaminess, and decline of living and working abilities. The “Pharmacopoeia of the People’s Republic of China” has recorded that *Bupleuri radix* tastes bitter, pungent, and microcold, belonging to the liver, gallbladder, and lung meridians. It relieves exterior and antipyretic effects, lifting Yang Qi and soothing liver-qi stagnation (27). *Bupleuri radix* was first recorded in Sheng Nong’s herbal classic: “*Bupleuri radix*, mainly to the heart and abdomen, removes the Qi in the intestines and stomach, accumulates food, cold, and heat pathogens, and eliminates the old to bring forth the new. Take it for a long time, lighten your body, brighten your eyes, and improve your essence.” Modern pharmacological studies have shown that *Bupleuri radix* has sedative, antipyretic, analgesic, anti-inflammatory, immune-enhancing, antidepressant, and antitumor effects (28). *Paeoniae Radix Alba* was first recorded in Sheng Nong’s herbal classic. The medicine tastes bitter, sour, and slightly cold and belongs to the meridians of the liver and spleen. It nourishes blood, regulates meridians, retains Yin, stops sweat, softens the liver, relieves pain, and represses the liver (29). Modern research has shown that *Paeoniae Radix Alba* has analgesic, anti-inflammatory, liver-protective, antioxidant, and other effects (30–33). It has been claimed that the drug combination of Radix Bupleuri and Radix Paeoniae Alba can greatly lessen melancholy brought on by Chronic Unpredictable Mild Stress (CUMS). The hypothalamic–pituitary–adrenal (HPA) axis is regulated, neurotoxicity is inhibited, and the production of brain-derived neurotrophic factors is controlled (9). The desiccated root of *Pueraria lobata* is known as *Puerariae radix*. In an ancient book titled “Treatise on febrile and miscellaneous diseases,” it was noted that *Pueraria lobata* had significant benefits for easing muscles, bringing down temperature, and reviving and alleviating diarrhea.” According to the “Compendium of Materia Medica,” *Pueraria lobata* has cooling, calming, and pleasant tastes as well as purifying and heat-clearing properties. *Pueraria lobata* can be used to treat fever, thirst, severe dysentery, diarrhea, diabetes, and hypertension, according to the 2020 version of the “Pharmacopoeia of the People’s Republic of China”. *Puerariae Radix* and its compounds have been shown by studies in contemporary science and medicine to have antioxidant, antihypertension, anticancer, antidiabetes/nephropathy, and neuroprotective properties (34–38). These studies were aimed at determining the mechanism of a single ingredient. The curative effect of TCM depends on the joint action of various effective components, which play a role through multiple links, targets, and steps.

Network pharmacology can reveal the numerous components, targets, and pathways that make up TCM’s mechanism. The curative effect of TCM depends on the joint action of various effective ingredients. As a result, network pharmacology is frequently used to investigate how TCM works to cure melancholy. For instance, Wang G et al. used network pharmacology to identify 52 biological processes and 35 signaling pathways associated with the antidepressant effect of *Puerariae Radix*. They also obtained eight major active components, 64 potential antidepressant gene targets, and 15 core antidepressant gene targets of *Puerariae Radix* (8).

In this work, the TCMSP database yielded the identification of 260 active substances. A total of 153 targets that were designated “key targets” for PPI network building and GO-KEGG analysis were obtained by using a Venn tool to connect the Chaihu Anxin Capsule targets and depression targets. These 153 important targets were uploaded to the STRING database and used to build the PPI network. IL6, IL1B, AKT1, TP53, and STAT3 were deemed important targets implicated in the effects of Chaihu Anxin capsule on depression and had high degree values in the PPI network. The AGE-RAGE signaling pathway in diabetic complications and lipids and atherosclerosis were the most enriched pathways, according to GO and KEGG enrichment studies of the 153 important targets. After molecular docking, quercetin, beta-sitosterol and kaempferol were discovered to interact directly with IL6, IL1B, AKT1, TP53, and STAT3.

The quercetin, beta-sitosterol, and kaempferol components were found through network analysis to be the most promising ingredients. As reported, quercetin could reduce lipopolysaccharide-induced depressive-like behaviors and impairments in learning and memory in rats. The process underlying this effect may involve controlling the expression of Copine 6 and triggering receptors expressed on myeloid cells (TREM) 1/2, which are linked to brain derived neurotrophic factor (BDNF), in the prefrontal cortex (PFC) and hippocampus (39). Yin Y et al. found that beta-sitosterol and its derivatives have effects that are similar to those of antidepressants on male mature rodents, and these effects are mediated by the 5-hydroxytryptamine (5-HT), Dopamine (DA), and Gamma-aminobutyric acid (GABA)-ergic systems (40). A review suggested that kaempferol modulates several inflammation signaling pathways, including nuclear factor kappa B (NF-kB), p38 mitogen-activated protein kinases (p38MAPK), serine/threonine kinase (AKT), and the -catenin cascade, to exert its multipotential neuroprotective effects (41).

The PPI network identified the IL6, IL1B, AKT1, TP53, and STAT3 genes as significant targets. IL-6 is a peripheral or central cytokine that plays a significant role in stress responses and depressive disorders, particularly physical illnesses that coexist with depression, according to evidence from animal and human studies (42). In the central nervous system, IL-1β is released by several cells that mediate inflammation, plays a role in central nervous system development, and participates in neural growth and synaptic organization. IL-1β overexpression has been associated with exacerbated neuroinflammatory reactions as well as impaired neurotransmission and cellular metabolism. All of these processes may contribute to pathological events associated with depression (43). Studies in animal models have shown that IL-1β injection produces depressive-like symptoms, such as anhedonia, weight loss, fatigue, and impaired social interaction (44). AKT1, a downstream enzyme that has been linked to the pathogenesis of neurotransmitter-related diseases such as melancholy, has been shown to exist (45). TP53 pathways have been shown to be the most distinct pathways linked to major depressive disorder caused by inflammation (46). Interactions between neurons and microglia are essential for sustaining the nervous system, and the balance of the nervous system is crucial for understanding the pathophysiology of depression. The effects of microglial-derived synaptic alterations on eliciting antidepressant-like behavior were investigated using microglia-specific STAT3 knockout mice. The findings of the FST, TST, sucrose preference, and open field tests revealed that microglia-specific STAT3 deletion animals had antidepressant-like behavior (46).

The 153 possible targets were subjected to GO enrichment analysis and KEGG enrichment analysis. The findings of the GO analysis suggested that cellular response to nitrogen compound, xenobiotic stimulus, molecule of bacterial origin, neurotransmitter receptor activity, and postsynaptic membrane may play a role in the treatment of depression. These targets were mostly connected to lipids and atherosclerosis as well as the AGE-RAGE signaling pathway in diabetic complications, according to KEGG pathway analysis. The most significant cardiovascular risk factors for a poor prognosis in myocardial infarction patients now include chronic depression. In a review, the importance of the central and autonomic control of cardiac functions, or the neuro-cardiac axis, is thoroughly explained, highlighting the functions of acute and chronic stress, circadian rhythms, emotions and the social environment in initiating acute cardiac events and deteriorating heart function and metabolism in chronic cardiovascular diseases (47). The AGE-RAGE pathway has a role in a number of pathological situations, such as cancer, diabetes, cardiovascular disease, and neurodegenerative disorders. Proinflammatory cytokines (including interleukin-1, interleukin-6, and tumor necrosis factor-α), growth factors such as vascular endothelial growth factor (VEGF), and NF-kB activation are all mediated through the AGE-RAGE signaling pathway, which promotes the onset and severity of depression (48, 49). Inflammation is a critical disease modifier that promotes susceptibility to depression. As a key disease modulator that increases vulnerability to depression, inflammation is a major health issue. As a result of early life trauma, a more pronounced stress response, microbiome changes, hereditary disorders, or a combination of these and other variables, controlling inflammation can have a therapeutic impact overall. As shown in the KEGG pathway diagram, the targets included in the lipid and atherosclerosis pathways mainly included AKT, STAT3, IL-1β, IL-6, and TP53. Wang R (50) and other researchers found that genes related to atherosclerosis and endothelial glycolysis include AKT1, IL-6, VEGFA, TP53, STAT3, SRC and MAPK1. These genes play a key role in atherosclerosis by regulating multiple signaling pathways related to cell signal transduction, energy metabolism, immune function and blood clot formation. In the KEGG pathway map, the AGE-RAGE signaling pathway in diabetes complications includes AKT, IL-6, STAT3 and other targets, which all play an important regulatory role in the AGE-RAGE pathway.

Molecular docking results confirmed that quercetin, beta-sitosterol and kaempferol had good affinities for depression-related molecules, such as IL6, IL1B, AKT1, TP53, and STAT3. A review noted that quercetin could decrease the mRNA expression levels of IL1B (51). Selvaraj Jayaraman et al. showed that beta-sitosterol could restore the elevated serum levels of proinflammatory cytokines such as IL-6 (52). Feng Zhang et al. found that β-sitosterol significantly decreased the expression of STAT3 (53). Kaempferol was proven to decrease the protein expression of AKT and IL-6 and increase TP53 levels (54).

However, this study has some drawbacks. More research is required to determine how Chaihu Anxin Capsule treats depression by activating the AGE-RAGE signaling pathway in diabetes complications as well as the lipid and atherosclerosis pathways. The mechanism of the Chaihu Anxin capsule identified in this study also requires more analysis and verification through experimental investigation. The effects and molecular mechanism of Chaihu Anxin Capsule in the treatment of depression will be further investigated using molecular biology techniques.

## 5 Conclusions

In conclusion, a total of 260 active ingredients were identified from the TCMSP database. The Chaihu Anxin capsule targets and depression targets were intersected by using a Venn tool to obtain 153 targets, identified as “key targets” for PPI network construction and GO\KEGG analysis. Targets with high degree values in the PPI network included IL6, IL1B, AKT1, TP53, and STAT3. These genes were considered significant targets involved in the effects of Chaihu Anxin capsule on depression. KEGG enrichment analyses of the 153 key targets revealed that lipid and atherosclerosis and the AGE-RAGE signaling pathway in diabetic complications were the most enriched pathways. After molecular docking, quercetin, beta-sitosterol and kaempferol were found to interact directly with IL6, IL1B, AKT1, TP53, and STAT3. Our study elaborated the multicomponent synergy mechanisms of the Chaihu Anxin capsule in the treatment of depression for the first time, which also provided a pharmacological basis for treating depression.

## Data availability statement

The datasets presented in this study can be found in online repositories. The names of the repository/repositories and accession number(s) can be found in the article/**Supplementary Material**.

## Author contributions

LY: Data curation, Methodology, Software, Visualization, Writing – original draft. YZ: Data curation, Formal Analysis, Investigation, Software, Supervision, Validation, Writing – original draft. RQ: Conceptualization, Investigation, Methodology, Writing – original draft. YF: Conceptualization, Methodology, Validation, Writing – review & editing. CZ: Funding acquisition, Resources, Supervision, Writing – review & editing. JY: Funding acquisition, Project administration, Resources, Validation, Writing – review & editing.

## References

[1] McCarron RM Shapiro B Rawles J Luo J. Depression. Ann Intern Med (2021) 174:ITC65–80. doi: 10.7326/AITC202105180 33971098

[2] Beurel E Toups M Nemeroff CB. The bidirectional relationship of depression and inflammation: double trouble. Neuron (2020) 107:234–56. doi: 10.1016/j.neuron.2020.06.002 PMC7381373 32553197

[3] Malhi GS Mann JJ. Depression. Lancet (2018) 392:2299–312. doi: 10.1016/S0140-6736(18)31948-2 30396512

[4] Wang YS Shen CY Jiang JG. Antidepressant active ingredients from herbs and nutraceuticals used in TCM: pharmacological mechanisms and prospects for drug discovery. Pharmacol Res (2019) 150:104520. doi: 10.1016/j.phrs.2019.104520 31706012

[5] Wang Y Peng M. Research progress on classical traditional chinese medicine jieyu pills in the treatment of depression. Neuropsychiatr Dis Treat (2020) 16:3023–33. doi: 10.2147/NDT.S282384 PMC7733407 33324063

[6] Su R Fan J Li T Cao X Zhou J Han Z. Jiawei Xiaoyao capsule treatment for mild to moderate major depression with anxiety symptoms: a randomized, double-blind, double-dummy, controlled, multicenter, parallel-treatment trial. J Tradit Chin Med (2019) 39:410–7. 32186013

[7] Zhang M Bai X. Shugan jieyu capsule in post-stroke depression treatment: from molecules to systems. Front Pharmacol (2022) 13:821270. doi: 10.3389/fphar.2022.821270 35140618 PMC8818889

[8] Wang G Luo P Zhang S Huang Q Zhang S Zeng Q. Screening and identification of antidepressant active ingredients from puerariae radix extract and study on its mechanism. Oxid Med Cell Longev (2021) 2021:2230195. doi: 10.1155/2021/2230195 34539968 PMC8445728

[9] Zhang H Zhang S Hu M Chen Y Wang W Zhang K. An integrative metabolomics and network pharmacology method for exploring the effect and mechanism of Radix Bupleuri and Radix Paeoniae Alba on anti-depression. J Pharm BioMed Anal (2020) 189:113435. doi: 10.1016/j.jpba.2020.113435 32653815

[10] Zhao YY Yu J Yang L Chen W Zhou C Wang L. Effect of Chaihu Anxin capsules on depression -like behavior and corticosterone in reserpine rats. Chin J Clin Pharmacol (2021) 37:3131–3134+3152. doi: 10.13699/j.cnki.1001-6821.2021.22.028

[11] Ru J Li P Wang J Zhou W Li B Huang C. TCMSP: a database of systems pharmacology for drug discovery from herbal medicines. J Cheminform (2014) 6:13. doi: 10.1186/1758-2946-6-13 24735618 PMC4001360

[12] Wang Y Sun Y Wang R Du J Wang Q. Network pharmacology and molecular docking analysis on the pharmacological mechanisms of modified sanmiaosan in treating ulcerative colitis. Comput Math Methods Med (2022) 2022:2556521. doi: 10.1155/2022/2556521 35966251 PMC9371879

[13] Yuan G Shi S Jia Q Shi J Shi S Zhang X. Use of network pharmacology to explore the mechanism of gegen (Puerariae lobatae radix) in the treatment of type 2 diabetes mellitus associated with hyperlipidemia. Evid Based Complement Alternat Med (2021) 2021:6633402. doi: 10.1155/2021/6633402 33953784 PMC8068526

[14] Yang L Yu J Zhou CH Wang LJ. Simultaneous determination of seven bioactive components of Chaihu-Anxin capsule in plasma by HPLC-MS/MS. Chin J Clin Pharmacol (2022) 38:2628–33. doi: 10.13699/j.cnki.1001-6821.2022.21.024

[15] UniProt Consortium. UniProt: a hub for protein information. Nucleic Acids Res (2015) 43:D204–12. doi: 10.1093/nar/gku989 PMC4384041 25348405

[16] Shannon P Markiel A Ozier O Baliga NS Wang JT Ramage D. Cytoscape: a software environment for integrated models of biomolecular interaction networks. Genome Res (2003) 13:2498–504. doi: 10.1101/gr.1239303 PMC403769 14597658

[17] Rebhan M Chalifa-Caspi V Prilusky J Lancet D. GeneCards: integrating information about genes, proteins and diseases. Trends Genet (1997) 13:163. doi: 10.1016/s0168-9525(97)01103-7 9097728

[18] Amberger JS Hamosh A. Searching online mendelian inheritance in man (OMIM): A knowledgebase of human genes and genetic phenotypes. Curr Protoc Bioinf (2017) 58:1.2.1–1.2.12. doi: 10.1002/cpbi.27 PMC5662200 28654725

[19] Wishart DS Feunang YD Guo AC Lo EJ Marcu A Grant JR. DrugBank 5.0: a major update to the DrugBank database for 2018. Nucleic Acids Res (2018) 46:D1074–82. doi: 10.1093/nar/gkx1037 PMC5753335 29126136

[20] Zhou Y Zhang Y Lian X Li F Wang C Zhu F. Therapeutic target database update 2022: facilitating drug discovery with enriched comparative data of targeted agents. Nucleic Acids Res (2022) 50:D1398–407. doi: 10.1093/nar/gkab953 PMC8728281 34718717

[21] Szklarczyk D Gable AL Lyon D Junge A Wyder S Huerta-Cepas J. STRING v11: protein-protein association networks with increased coverage, supporting functional discovery in genome-wide experimental datasets. Nucleic Acids Res (2019) 47:D607–13. doi: 10.1093/nar/gky1131 PMC6323986 30476243

[22] Zhou Y Zhou B Pache L Chang M Khodabakhshi AH Tanaseichuk O. Metascape provides a biologist-oriented resource for the analysis of systems-level datasets. Nat Commun (2019) 10:1523. doi: 10.1038/s41467-019-09234-6 30944313 PMC6447622

[23] Berman H Henrick K Nakamura H. Announcing the worldwide protein data bank. Nat Struct Biol (2003) 10:980. doi: 10.1038/nsb1203-980 14634627

[24] Kim S Chen J Cheng T Gindulyte A He J He S. PubChem in 2021: new data content and improved web interfaces. Nucleic Acids Res (2021) 49:D1388–95. doi: 10.1093/nar/gkaa971 PMC7778930 33151290

[25] Trott O Olson AJ. AutoDock Vina: improving the speed and accuracy of docking with a new scoring function, efficient optimization, and multithreading. J Comput Chem (2010) 31:455–61. doi: 10.1002/jcc.21334 PMC3041641 19499576

[26] Seeliger D de Groot BL. Ligand docking and binding site analysis with PyMOL and Autodock/Vina. J Comput Aided Mol Des (2010) 24:417–22. doi: 10.1007/s10822-010-9352-6 PMC2881210 20401516

[27] Commission CP. Pharmacopoeia of the people's republic of China (Volume I). Beijing: The Medicine Science and Technology Press of China. (2020) p. 293.

[28] Ashour ML Wink M. Genus Bupleurum: a review of its phytochemistry, pharmacology and modes of action. J Pharm Pharmacol (2011) 63:305–21. doi: 10.1111/j.2042-7158.2010.01170.x PMC7197585 21749378

[29] Xu JX Xu J Cao Y Zhu YJ Li XY Ge DZ. Modern research progress of traditional Chinese medicine Paeoniae Radix Alba and prediction of its Q-markers. Zhongguo Zhong Yao Za Zhi (2021) 46:5486–95. doi: 10.19540/j.cnki.cjcmm.20210818.201 34951200

[30] Xie T Li K Gong X Jiang R Huang W Chen X. Paeoniflorin protects against liver ischemia/reperfusion injury in mice *via* inhibiting HMGB1-TLR4 signaling pathway. Phytother Res (2018) 32:2247–55. doi: 10.1002/ptr.6161 30047580

[31] Liu B Lin J Bai L Zhou Y Lu R Zhang P. Paeoniflorin inhibits mesangial cell proliferation and inflammatory response in rats with mesangial proliferative glomerulonephritis through PI3K/AKT/GSK-3β Pathway. Front Pharmacol (2019) 10:978. doi: 10.3389/fphar.2019.00978 31551783 PMC6745507

[32] Jo GH Kim SN Kim MJ Heo Y. Protective effect of Paeoniae radix alba root extract on immune alterations in mice with atopic dermatitis. J Toxicol Environ Health A (2018) 81:502–11. doi: 10.1080/15287394.2018.1460785 29630468

[33] Li J Huang S Huang W Wang W Wen G Gao L. Paeoniflorin ameliorates interferon-alpha-induced neuroinflammation and depressive-like behaviors in mice. Oncotarget (2017) 8:8264–82. doi: 10.18632/oncotarget.14160 PMC5352399 28030814

[34] Zhou X Lam WP Tang HC Koon CM Cheng L Lau CB. Effects of Gegen (Puerariae lobatae Radix) water extract on improving detrusor overactivity in spontaneously hypertensive rats. Phytomedicine (2016) 23:672–8. doi: 10.1016/j.phymed.2016.03.002 27161408

[35] Zhang D Ma G Hou M Zhang T Chen L Zhao C. The neuroprotective effect of puerarin in acute spinal cord injury rats. Cell Physiol Biochem (2016) 39:1152–64. doi: 10.1159/000447822 27576607

[36] Yu WL Zhao YP Shu B. The radical scavenging activities of radix puerariae isoflavonoids: A chemiluminescence study. Food Chem (2004) 86:525–9. doi: 10.1016/j.foodchem.2003.09.005

[37] Shukla R Banerjee S Tripathi YB. Antioxidant and Antiapoptotic effect of aqueous extract of Pueraria tuberosa (Roxb. Ex Willd.) DC. On streptozotocin-induced diabetic nephropathy in rats. BMC Complement Altern Med (2018) 18:156. doi: 10.1186/s12906-018-2221-x 29751837 PMC5948837

[38] Wu XY Chen GL Wang YL Li CL Bi L Chen Y. Mechanism of traditional chinese medicine in treatment of immune thrombocytopenia. Chin J Exp Traditional Med Formulae (2017) 23:213–9. doi: 10.13422/j.cnki.syfjx.2017080213

[39] Fang K Li HR Chen XX Gao XR Huang LL Du AQ. Quercetin alleviates LPS-induced depression-like behavior in rats *via* regulating BDNF-related imbalance of copine 6 and TREM1/2 in the hippocampus and PFC. Front Pharmacol (2020) 10:1544. doi: 10.3389/fphar.2019.01544 32009956 PMC6978986

[40] Yin Y Liu X Liu J Cai E Zhao Y Li H. The effect of beta-sitosterol and its derivatives on depression by the modification of 5-HT, DA and GABA-ergic systems in mice. RSC Adv (2018) 8(2):671–80. doi: 10.1039/c7ra11364a PMC9076981 35538977

[41] Silva Dos Santos J Gonçalves Cirino JP de Oliveira Carvalho P Ortega MM. The pharmacological action of kaempferol in central nervous system diseases: A review. Front Pharmacol (2021) 11:565700. doi: 10.3389/fphar.2020.565700 33519431 PMC7838523

[42] Ting EY Yang AC Tsai SJ. Role of interleukin-6 in depressive disorder. Int J Mol Sci (2020) 21:2194. doi: 10.3390/ijms21062194 32235786 PMC7139933

[43] Ferreira AM Leal B Ferreira I Brás S Moreira I Samões R. Depression and anxiety in multiple sclerosis patients: The role of genetic variability of interleukin 1β. Mult Scler Relat Disord (2021) 52:102982. doi: 10.1016/j.msard.2021.102982 34004436

[44] Dinarello CA. Therapeutic strategies to reduce IL-1 activity in treating local and systemic inflammation. Curr Opin Pharmacol (2004) 4:378–85. doi: 10.1016/j.coph.2004.03.010 15251132

[45] Pereira PA Bicalho MA de Moraes EN Malloy-Diniz L Bozzi IC Nicolato R. Genetic variant of AKT1 and AKTIP associated with late-onset depression in a Brazilian population. Int J Geriatr Psychiatry (2014) 29:399–405. doi: 10.1002/gps.4018 24022875

[46] Moisan MP Foury A Dexpert S Cole SW Beau C Forestier D. Transcriptomic signaling pathways involved in a naturalistic model of inflammation-related depression and its remission. Transl Psychiatry (2021) 11:203. doi: 10.1038/s41398-021-01323-9 33824279 PMC8024399

[47] Fioranelli M Bottaccioli AG Bottaccioli F Bianchi M Rovesti M Roccia MG. Stress and inflammation in coronary artery disease: A review psychoneuroendocrineimmunology-based. Front Immunol (2018) 9:2031. doi: 10.3389/fimmu.2018.02031 30237802 PMC6135895

[48] Yu XD Zhang D Xiao CL Zhou Y Li X Wang L. P-coumaric acid reverses depression-like behavior and memory deficit *via* inhibiting AGE-RAGE-mediated neuroinflammation. Cells (2022) 11:1594. doi: 10.3390/cells11101594 35626632 PMC9139330

[49] Hudson BI Lippman ME. Targeting RAGE signaling in inflammatory disease. Annu Rev Med (2018) 69:349–64. doi: 10.1146/annurev-med-041316-085215 29106804

[50] Wang R Wang M Ye J Sun G Sun X. Mechanism overview and target mining of atherosclerosis: Endothelial cell injury in atherosclerosis is regulated by glycolysis (Review). Int J Mol Med (2021) 47:65–76. doi: 10.3892/ijmm.2020.4798 PMC7723681 33236132

[51] Wu Y Zhu Y Xie N Wang H Wang F Zhou J. A network pharmacology approach to explore active compounds and pharmacological mechanisms of a patented Chinese herbal medicine in the treatment of endometriosis. PloS One (2022) 17:e0263614. doi: 10.1371/journal.pone.0263614 35130311 PMC8820622

[52] Jayaraman S Devarajan N Rajagopal P Babu S Ganesan SK Veeraraghavan VP. β-sitosterol circumvents obesity induced inflammation and insulin resistance by down-regulating IKKβ/NF-κB and JNK signaling pathway in adipocytes of type 2 diabetic rats. Molecules (2021) 26:2101. doi: 10.3390/molecules26072101 33917607 PMC8038823

[53] Zhang F Liu Z He X Li Z Shi B Cai F. β-Sitosterol-loaded solid lipid nanoparticles ameliorate complete Freund's adjuvant-induced arthritis in rats: involvement of NF-кB and HO-1/Nrf-2 pathway. Drug Delivery (2020) 27:1329–41. doi: 10.1080/10717544.2020.1818883 PMC7534215 32945205

[54] Chen Z Lin T Liao X Li Z Lin R Qi X. Network pharmacology based research into the effect and mechanism of Yinchenhao Decoction against Cholangiocarcinoma. Chin Med (2021) 16:13. doi: 10.1186/s13020-021-00423-4 33478536 PMC7818939

