# Supplementary material for: A network pharmacology and molecular docking approach to reveal the mechanism of Chaihu Anxin Capsule in depression
Source: Front Endocrinol (Lausanne). 2023 Sep 7;14:1256045. doi: 10.3389/fendo.2023.1256045 (PMC10513492; doi:10.3389/fendo.2023.1256045)
Supplement: Supplementary file 1 [file DataSheet_1.docx]

Supplementary Material

# Supplementary Tables

Supplementary Table 1: Active ingredients of Chaihu Anxin Capsule.

| Herbal | Mol ID | Molecule name | OB | DL |
| --- | --- | --- | --- | --- |
| *Paeoniae Radix Alba* | MOL001921 | Lactiflorin | 49.12 | 0.80 |
| *Paeoniae Radix Alba* | MOL001924 | paeoniflorin | 53.87 | 0.79 |
| *Paeoniae Radix Alba* | MOL000211 | Mairin | 55.38 | 0.78 |
| *Paeoniae Radix Alba* | MOL000358 | beta-sitosterol | 36.91 | 0.75 |
| *Paeoniae Radix Alba* | MOL000359 | sitosterol | 36.91 | 0.75 |
| *Paeoniae Radix Alba* | MOL001930 | benzoyl paeoniflorin | 31.27 | 0.75 |
| *Paeoniae Radix Alba* | MOL001919 | (3S,5R,8R,9R,10S,14S)-3,17-dihydroxy-4,4,8,10,14-pentamethyl-2,3,5,6,7,9-hexahydro-1H-cyclopenta[a]phenanthrene-15,16-dione | 43.56 | 0.53 |
| *Paeoniae Radix Alba* | MOL001925 | paeoniflorin_qt | 68.18 | 0.40 |
| *Paeoniae Radix Alba* | MOL001910 | 11alpha,12alpha-epoxy-3beta-23-dihydroxy-30-norolean-20-en-28,12beta-olide | 64.77 | 0.38 |
| *Paeoniae Radix Alba* | MOL001918 | paeoniflorgenone | 87.59 | 0.37 |
| *Paeoniae Radix Alba* | MOL001928 | albiflorin_qt | 66.64 | 0.33 |
| *Paeoniae Radix Alba* | MOL000492 | (+)-catechin | 54.83 | 0.24 |
| *Paeoniae Radix Alba* | MOL000422 | kaempferol | 41.88 | 0.24 |
| *Paeoniae Radix Alba* | MOL000513 | 3,4,5-trihydroxybenzoic acid | 31.69 | 0.04 |
| *Arum Ternatum Thunb* | MOL002670 | Cavidine | 35.64 | 0.81 |
| *Arum Ternatum Thunb* | MOL003578 | Cycloartenol | 38.69 | 0.78 |
| *Arum Ternatum Thunb* | MOL001755 | 24-Ethylcholest-4-en-3-one | 36.08 | 0.76 |
| *Arum Ternatum Thunb* | MOL000449 | Stigmasterol | 43.83 | 0.76 |
| *Arum Ternatum Thunb* | MOL002776 | Baicalin | 40.12 | 0.75 |
| *Arum Ternatum Thunb* | MOL000358 | beta-sitosterol | 36.91 | 0.75 |
| *Arum Ternatum Thunb* | MOL000519 | coniferin | 31.11 | 0.32 |
| *Arum Ternatum Thunb* | MOL006957 | (3S,6S)-3-(benzyl)-6-(4-hydroxybenzyl)piperazine-2,5-quinone | 46.89 | 0.27 |
| *Arum Ternatum Thunb* | MOL006937 | 12,13-epoxy-9-hydroxynonadeca-7,10-dienoic acid | 42.15 | 0.24 |
| *Arum Ternatum Thunb* | MOL002714 | baicalein | 33.52 | 0.21 |
| *Arum Ternatum Thunb* | MOL006967 | beta-D-Ribofuranoside, xanthine-9 | 44.72 | 0.21 |
| *Arum Ternatum Thunb* | MOL006936 | 10,13-eicosadienoic | 39.99 | 0.20 |
| *Arum Ternatum Thunb* | MOL005030 | gondoic acid | 30.70 | 0.20 |
| *Radix Bupleuri* | MOL000449 | Stigmasterol | 43.83 | 0.76 |
| *Radix Bupleuri* | MOL004718 | 伪-spinasterol | 42.98 | 0.76 |
| *Radix Bupleuri* | MOL002776 | Baicalin | 40.12 | 0.75 |
| *Radix Bupleuri* | MOL004653 | (+)-Anomalin | 46.06 | 0.66 |
| *Radix Bupleuri* | MOL013187 | Cubebin | 57.13 | 0.64 |
| *Radix Bupleuri* | MOL004702 | saikosaponin c_qt | 30.50 | 0.63 |
| *Radix Bupleuri* | MOL004598 | 3,5,6,7-tetramethoxy-2-(3,4,5-trimethoxyphenyl)chromone | 31.97 | 0.59 |
| *Radix Bupleuri* | MOL004624 | Longikaurin A | 47.72 | 0.53 |
| *Radix Bupleuri* | MOL004609 | Areapillin | 48.96 | 0.41 |
| *Radix Bupleuri* | MOL000354 | isorhamnetin | 49.60 | 0.31 |
| *Radix Bupleuri* | MOL000490 | petunidin | 30.05 | 0.31 |
| *Radix Bupleuri* | MOL004628 | Octalupine | 47.82 | 0.28 |
| *Radix Bupleuri* | MOL000098 | quercetin | 46.43 | 0.28 |
| *Radix Bupleuri* | MOL004648 | Troxerutin | 31.60 | 0.28 |
| *Radix Bupleuri* | MOL000422 | kaempferol | 41.88 | 0.24 |
| *Radix Bupleuri* | MOL004644 | Sainfuran | 79.91 | 0.23 |
| *Radix Bupleuri* | MOL001645 | Linoleyl acetate | 42.10 | 0.20 |
| *Radix Bupleuri* | MOL004637 | Saikosaponin D | 34.39 | 0.09 |
| *Radix Bupleuri* | MOL004635 | saikosaponin a | 32.39 | 0.09 |
| *Jujubae Fructus* | MOL012981 | Daechuine S7 | 44.82 | 0.83 |
| *Jujubae Fructus* | MOL000787 | Fumarine | 59.26 | 0.83 |
| *Jujubae Fructus* | MOL012980 | Daechuine S6 | 46.48 | 0.79 |
| *Jujubae Fructus* | MOL013357 | (3S,6R,8S,9S,10R,13R,14S,17R)-17-[(1R,4R)-4-ethyl-1,5-dimethylhexyl]-10,13-dimethyl-2,3,6,7,8,9,11,12,14,15,16,17-dodecahydro-1H-cyclopenta[a]phenanthrene-3,6-diol | 34.37 | 0.78 |
| *Jujubae Fructus* | MOL001454 | berberine | 36.86 | 0.78 |
| *Jujubae Fructus* | MOL000211 | Mairin | 55.38 | 0.78 |
| *Jujubae Fructus* | MOL008034 | 21302-79-4 | 73.52 | 0.77 |
| *Jujubae Fructus* | MOL004350 | Ruvoside_qt | 36.12 | 0.76 |
| *Jujubae Fructus* | MOL000449 | Stigmasterol | 43.83 | 0.76 |
| *Jujubae Fructus* | MOL000358 | beta-sitosterol | 36.91 | 0.75 |
| *Jujubae Fructus* | MOL012986 | Jujubasaponin V_qt | 36.99 | 0.63 |
| *Jujubae Fructus* | MOL005360 | malkangunin | 57.71 | 0.63 |
| *Jujubae Fructus* | MOL003410 | Ziziphin_qt | 66.95 | 0.62 |
| *Jujubae Fructus* | MOL012946 | zizyphus saponin I_qt | 32.69 | 0.62 |
| *Jujubae Fructus* | MOL012961 | jujuboside A_qt | 36.67 | 0.62 |
| *Jujubae Fructus* | MOL012989 | Jujuboside C_qt | 40.26 | 0.62 |
| *Jujubae Fructus* | MOL002773 | beta-carotene | 37.18 | 0.58 |
| *Jujubae Fructus* | MOL000783 | Protoporphyrin | 30.86 | 0.56 |
| *Jujubae Fructus* | MOL000627 | Stepholidine | 33.11 | 0.54 |
| *Jujubae Fructus* | MOL012992 | Mauritine D | 89.13 | 0.45 |
| *Jujubae Fructus* | MOL007213 | Nuciferin | 34.43 | 0.40 |
| *Jujubae Fructus* | MOL012976 | coumestrol | 32.49 | 0.34 |
| *Jujubae Fructus* | MOL012921 | stepharine | 31.55 | 0.33 |
| *Jujubae Fructus* | MOL000098 | quercetin | 46.43 | 0.28 |
| *Jujubae Fructus* | MOL008647 | Moupinamide | 86.71 | 0.26 |
| *Jujubae Fructus* | MOL000492 | (+)-catechin | 54.83 | 0.24 |
| *Jujubae Fructus* | MOL000096 | (-)-catechin | 49.68 | 0.24 |
| *Jujubae Fructus* | MOL001522 | (S)-Coclaurine | 42.35 | 0.24 |
| *Poria Cocos* | MOL000300 | dehydroeburicoic acid | 44.17 | 0.83 |
| *Poria Cocos* | MOL000285 | (2R)-2-[(5R,10S,13R,14R,16R,17R)-16-hydroxy-3-keto-4,4,10,13,14-pentamethyl-1,2,5,6,12,15,16,17-octahydrocyclopenta[a]phenanthren-17-yl]-5-isopropyl-hex-5-enoic acid | 38.26 | 0.82 |
| *Poria Cocos* | MOL000280 | (2R)-2-[(3S,5R,10S,13R,14R,16R,17R)-3,16-dihydroxy-4,4,10,13,14-pentamethyl-2,3,5,6,12,15,16,17-octahydro-1H-cyclopenta[a]phenanthren-17-yl]-5-isopropyl-hex-5-enoic acid | 31.07 | 0.82 |
| *Poria Cocos* | MOL000283 | Ergosterol peroxide | 40.36 | 0.81 |
| *Poria Cocos* | MOL000287 | 3beta-Hydroxy-24-methylene-8-lanostene-21-oic acid | 38.70 | 0.81 |
| *Poria Cocos* | MOL000276 | 7,9(11)-dehydropachymic acid | 35.11 | 0.81 |
| *Poria Cocos* | MOL000289 | pachymic acid | 33.63 | 0.81 |
| *Poria Cocos* | MOL000273 | (2R)-2-[(3S,5R,10S,13R,14R,16R,17R)-3,16-dihydroxy-4,4,10,13,14-pentamethyl-2,3,5,6,12,15,16,17-octahydro-1H-cyclopenta[a]phenanthren-17-yl]-6-methylhept-5-enoic acid | 30.93 | 0.81 |
| *Poria Cocos* | MOL000275 | trametenolic acid | 38.71 | 0.80 |
| *Poria Cocos* | MOL000279 | Cerevisterol | 37.96 | 0.77 |
| *Poria Cocos* | MOL000290 | Poricoic acid A | 30.61 | 0.76 |
| *Poria Cocos* | MOL000292 | poricoic acid C | 38.15 | 0.75 |
| *Poria Cocos* | MOL000296 | hederagenin | 36.91 | 0.75 |
| *Poria Cocos* | MOL000291 | Poricoic acid B | 30.52 | 0.75 |
| *Poria Cocos* | MOL000282 | ergosta-7,22E-dien-3beta-ol | 43.51 | 0.72 |
| *licorice* | MOL004924 | (-)-Medicocarpin | 40.99 | 0.95 |
| *licorice* | MOL004988 | Kanzonol F | 32.47 | 0.89 |
| *licorice* | MOL005018 | Xambioona | 54.85 | 0.87 |
| *licorice* | MOL004948 | Isoglycyrol | 44.70 | 0.84 |
| *licorice* | MOL004917 | glycyroside | 37.25 | 0.79 |
| *licorice* | MOL005001 | Gancaonin H | 50.10 | 0.78 |
| *licorice* | MOL000211 | Mairin | 55.38 | 0.78 |
| *licorice* | MOL000359 | sitosterol | 36.91 | 0.75 |
| *licorice* | MOL004903 | liquiritin | 65.69 | 0.74 |
| *licorice* | MOL004891 | shinpterocarpin | 80.30 | 0.73 |
| *licorice* | MOL004805 | (2S)-2-[4-hydroxy-3-(3-methylbut-2-enyl)phenyl]-8,8-dimethyl-2,3-dihydropyrano[2,3-f]chromen-4-one | 31.79 | 0.72 |
| *licorice* | MOL005013 | 18α-hydroxyglycyrrhetic acid | 41.16 | 0.71 |
| *licorice* | MOL002311 | Glycyrol | 90.78 | 0.67 |
| *licorice* | MOL004904 | licopyranocoumarin | 80.36 | 0.65 |
| *licorice* | MOL004959 | 1-Methoxyphaseollidin | 69.98 | 0.64 |
| *licorice* | MOL004824 | (2S)-6-(2,4-dihydroxyphenyl)-2-(2-hydroxypropan-2-yl)-4-methoxy-2,3-dihydrofuro[3,2-g]chromen-7-one | 60.25 | 0.63 |
| *licorice* | MOL005008 | Glycyrrhiza flavonol A | 41.28 | 0.60 |
| *licorice* | MOL005007 | Glyasperins M | 72.67 | 0.59 |
| *licorice* | MOL005003 | Licoagrocarpin | 58.81 | 0.58 |
| *licorice* | MOL005017 | Phaseol | 78.77 | 0.58 |
| *licorice* | MOL004966 | 3'-Hydroxy-4'-O-Methylglabridin | 43.71 | 0.57 |
| *licorice* | MOL004974 | 3'-Methoxyglabridin | 46.16 | 0.57 |
| *licorice* | MOL004806 | euchrenone | 30.29 | 0.57 |
| *licorice* | MOL004827 | Semilicoisoflavone B | 48.78 | 0.55 |
| *licorice* | MOL004905 | 3,22-Dihydroxy-11-oxo-delta(12)-oleanene-27-alpha-methoxycarbonyl-29-oic acid | 34.32 | 0.55 |
| *licorice* | MOL004884 | Licoisoflavone B | 38.93 | 0.55 |
| *licorice* | MOL004885 | licoisoflavanone | 52.47 | 0.54 |
| *licorice* | MOL001484 | Inermine | 75.18 | 0.54 |
| *licorice* | MOL004810 | glyasperin F | 75.84 | 0.54 |
| *licorice* | MOL004914 | 1,3-dihydroxy-8,9-dimethoxy-6-benzofurano[3,2-c]chromenone | 62.90 | 0.53 |
| *licorice* | MOL004978 | 2-[(3R)-8,8-dimethyl-3,4-dihydro-2H-pyrano[6,5-f]chromen-3-yl]-5-methoxyphenol | 36.21 | 0.52 |
| *licorice* | MOL004820 | kanzonols W | 50.48 | 0.52 |
| *licorice* | MOL004912 | Glabrone | 52.51 | 0.50 |
| *licorice* | MOL005012 | Licoagroisoflavone | 57.28 | 0.49 |
| *licorice* | MOL004879 | Glycyrin | 52.61 | 0.47 |
| *licorice* | MOL004855 | Licoricone | 63.58 | 0.47 |
| *licorice* | MOL004908 | Glabridin | 53.25 | 0.47 |
| *licorice* | MOL004857 | Gancaonin B | 48.79 | 0.45 |
| *licorice* | MOL004833 | Phaseolinisoflavan | 32.01 | 0.45 |
| *licorice* | MOL004911 | Glabrene | 46.27 | 0.44 |
| *licorice* | MOL004808 | glyasperin B | 65.22 | 0.44 |
| *licorice* | MOL004849 | 3-(2,4-dihydroxyphenyl)-8-(1,1-dimethylprop-2-enyl)-7-hydroxy-5-methoxy-coumarin | 59.62 | 0.43 |
| *licorice* | MOL004913 | 1,3-dihydroxy-9-methoxy-6-benzofurano[3,2-c]chromenone | 48.14 | 0.43 |
| *licorice* | MOL004814 | Isotrifoliol | 31.94 | 0.42 |
| *licorice* | MOL004949 | Isolicoflavonol | 45.17 | 0.42 |
| *licorice* | MOL004883 | Licoisoflavone | 41.61 | 0.42 |
| *licorice* | MOL004866 | 2-(3,4-dihydroxyphenyl)-5,7-dihydroxy-6-(3-methylbut-2-enyl)chromone | 44.15 | 0.41 |
| *licorice* | MOL004935 | Sigmoidin-B | 34.88 | 0.41 |
| *licorice* | MOL004863 | 3-(3,4-dihydroxyphenyl)-5,7-dihydroxy-8-(3-methylbut-2-enyl)chromone | 66.37 | 0.41 |
| *licorice* | MOL004989 | 6-prenylated eriodictyol | 39.22 | 0.41 |
| *licorice* | MOL004864 | 5,7-dihydroxy-3-(4-methoxyphenyl)-8-(3-methylbut-2-enyl)chromone | 30.49 | 0.41 |
| *licorice* | MOL004993 | 8-prenylated eriodictyol | 53.79 | 0.40 |
| *licorice* | MOL004856 | Gancaonin A | 51.08 | 0.40 |
| *licorice* | MOL004811 | Glyasperin C | 45.56 | 0.40 |
| *licorice* | MOL005000 | Gancaonin G | 60.44 | 0.39 |
| *licorice* | MOL004838 | 8-(6-hydroxy-2-benzofuranyl)-2,2-dimethyl-5-chromenol | 58.44 | 0.38 |
| *licorice* | MOL004915 | Eurycarpin A | 43.28 | 0.37 |
| *licorice* | MOL005020 | dehydroglyasperins C | 53.82 | 0.37 |
| *licorice* | MOL003656 | Lupiwighteone | 51.64 | 0.37 |
| *licorice* | MOL004882 | Licocoumarone | 33.21 | 0.36 |
| *licorice* | MOL004907 | Glyzaglabrin | 61.07 | 0.35 |
| *licorice* | MOL004815 | (E)-1-(2,4-dihydroxyphenyl)-3-(2,2-dimethylchromen-6-yl)prop-2-en-1-one | 39.62 | 0.35 |
| *licorice* | MOL004828 | Glepidotin A | 44.72 | 0.35 |
| *licorice* | MOL004829 | Glepidotin B | 64.46 | 0.34 |
| *licorice* | MOL002565 | Medicarpin | 49.22 | 0.34 |
| *licorice* | MOL004961 | Quercetin der. | 46.45 | 0.33 |
| *licorice* | MOL004980 | Inflacoumarin A | 39.71 | 0.33 |
| *licorice* | MOL004848 | licochalcone G | 49.25 | 0.32 |
| *licorice* | MOL004945 | (2S)-7-hydroxy-2-(4-hydroxyphenyl)-8-(3-methylbut-2-enyl)chroman-4-one | 36.57 | 0.32 |
| *licorice* | MOL004910 | Glabranin | 52.90 | 0.31 |
| *licorice* | MOL004898 | (E)-3-[3,4-dihydroxy-5-(3-methylbut-2-enyl)phenyl]-1-(2,4-dihydroxyphenyl)prop-2-en-1-one | 46.27 | 0.31 |
| *licorice* | MOL000354 | isorhamnetin | 49.60 | 0.31 |
| *licorice* | MOL005016 | Odoratin | 49.95 | 0.30 |
| *licorice* | MOL000239 | Jaranol | 50.83 | 0.29 |
| *licorice* | MOL000497 | licochalcone a | 40.79 | 0.29 |
| *licorice* | MOL000098 | quercetin | 46.43 | 0.28 |
| *licorice* | MOL004860 | licorice glycoside E | 32.89 | 0.27 |
| *licorice* | MOL004990 | 7,2',4'-trihydroxy－5-methoxy-3－arylcoumarin | 83.71 | 0.27 |
| *licorice* | MOL004991 | 7-Acetoxy-2-methylisoflavone | 38.92 | 0.26 |
| *licorice* | MOL000417 | Calycosin | 47.75 | 0.24 |
| *licorice* | MOL000422 | kaempferol | 41.88 | 0.24 |
| *licorice* | MOL000392 | formononetin | 69.67 | 0.21 |
| *licorice* | MOL004328 | naringenin | 59.29 | 0.21 |
| *licorice* | MOL004957 | HMO | 38.37 | 0.21 |
| *licorice* | MOL000500 | Vestitol | 74.66 | 0.21 |
| *licorice* | MOL003896 | 7-Methoxy-2-methyl isoflavone | 42.56 | 0.20 |
| *licorice* | MOL004985 | icos-5-enoic acid | 30.70 | 0.20 |
| *licorice* | MOL004996 | gadelaidic acid | 30.70 | 0.20 |
| *licorice* | MOL004841 | Licochalcone B | 76.76 | 0.19 |
| *licorice* | MOL004835 | Glypallichalcone | 61.60 | 0.19 |
| *licorice* | MOL001792 | DFV | 32.76 | 0.18 |
| *licorice* | MOL004941 | (2R)-7-hydroxy-2-(4-hydroxyphenyl)chroman-4-one | 71.12 | 0.18 |
| *Radix Puerariae* | MOL000358 | beta-sitosterol | 36.91 | 0.75 |
| *Radix Puerariae* | MOL012297 | puerarin | 24.03 | 0.69 |
| *Radix Puerariae* | MOL003629 | Daidzein-4,7-diglucoside | 47.27 | 0.67 |
| *Radix Puerariae* | MOL002959 | 3'-Methoxydaidzein | 48.57 | 0.24 |
| *Radix Puerariae* | MOL000392 | formononetin | 69.67 | 0.21 |
| *Radix Puerariae* | MOL000390 | daidzein | 19.44 | 0.19 |
| *Cinnamomi Ramulus* | MOL011169 | Peroxyergosterol | 44.39 | 0.82 |
| *Cinnamomi Ramulus* | MOL000358 | beta-sitosterol | 36.91 | 0.75 |
| *Cinnamomi Ramulus* | MOL000359 | sitosterol | 36.91 | 0.75 |
| *Cinnamomi Ramulus* | MOL004576 | taxifolin | 57.84 | 0.27 |
| *Cinnamomi Ramulus* | MOL001736 | (-)-taxifolin | 60.51 | 0.27 |
| *Cinnamomi Ramulus* | MOL000492 | (+)-catechin | 54.83 | 0.24 |
| *Cinnamomi Ramulus* | MOL000073 | ent-Epicatechin | 48.96 | 0.24 |
| *Zingiber Officinale Roscoe* | MOL000449 | Stigmasterol | 43.83 | 0.76 |
| *Zingiber Officinale Roscoe* | MOL000358 | beta-sitosterol | 36.91 | 0.75 |
| *Zingiber Officinale Roscoe* | MOL001771 | poriferast-5-en-3beta-ol | 36.91 | 0.75 |
| *Zingiber Officinale Roscoe* | MOL006129 | 6-methylgingediacetate2 | 48.73 | 0.32 |
| *Zingiber Officinale Roscoe* | MOL008698 | Dihydrocapsaicin | 47.07 | 0.19 |
| *Prunellae Spica* | MOL006772 | poriferasterol monoglucoside_qt | 43.83 | 0.76 |
| *Prunellae Spica* | MOL000449 | Stigmasterol | 43.83 | 0.76 |
| *Prunellae Spica* | MOL004355 | Spinasterol | 42.98 | 0.76 |
| *Prunellae Spica* | MOL006774 | stigmast-7-enol | 37.42 | 0.75 |
| *Prunellae Spica* | MOL000358 | beta-sitosterol | 36.91 | 0.75 |
| *Prunellae Spica* | MOL004798 | delphinidin | 40.63 | 0.28 |
| *Prunellae Spica* | MOL000098 | quercetin | 46.43 | 0.28 |
| *Prunellae Spica* | MOL000737 | morin | 46.23 | 0.27 |
| *Prunellae Spica* | MOL006767 | Vulgaxanthin-I | 56.14 | 0.26 |
| *Prunellae Spica* | MOL000006 | luteolin | 36.16 | 0.25 |
| *Prunellae Spica* | MOL000422 | kaempferol | 41.88 | 0.24 |
| *Prunellae Spica* | MOL000415 | rutin | 3.20 | 0.68 |
| *Corydalis Rhizoma* | MOL000791 | bicuculline | 69.67 | 0.88 |
| *Corydalis Rhizoma* | MOL001463 | Dihydrosanguinarine | 59.31 | 0.86 |
| *Corydalis Rhizoma* | MOL001474 | sanguinarine | 37.81 | 0.86 |
| *Corydalis Rhizoma* | MOL004231 | Tetrahydrocorysamine | 34.17 | 0.86 |
| *Corydalis Rhizoma* | MOL004225 | pseudocoptisine | 38.97 | 0.86 |
| *Corydalis Rhizoma* | MOL001458 | coptisine | 30.67 | 0.86 |
| *Corydalis Rhizoma* | MOL004230 | stylopine | 48.25 | 0.85 |
| *Corydalis Rhizoma* | MOL004198 | 18797-79-0 | 46.06 | 0.85 |
| *Corydalis Rhizoma* | MOL004226 | 24240-05-9 | 53.75 | 0.83 |
| *Corydalis Rhizoma* | MOL000787 | Fumarine | 59.26 | 0.83 |
| *Corydalis Rhizoma* | MOL001461 | Dihydrochelerythrine | 32.73 | 0.81 |
| *Corydalis Rhizoma* | MOL004202 | dehydrocavidine | 38.99 | 0.81 |
| *Corydalis Rhizoma* | MOL002670 | Cavidine | 35.64 | 0.81 |
| *Corydalis Rhizoma* | MOL004190 | (-)-alpha-N-methylcanadine | 45.06 | 0.80 |
| *Corydalis Rhizoma* | MOL004228 | saulatine | 42.74 | 0.79 |
| *Corydalis Rhizoma* | MOL001454 | berberine | 36.86 | 0.78 |
| *Corydalis Rhizoma* | MOL002903 | (R)-Canadine | 55.37 | 0.77 |
| *Corydalis Rhizoma* | MOL000449 | Stigmasterol | 43.83 | 0.76 |
| *Corydalis Rhizoma* | MOL000359 | sitosterol | 36.91 | 0.75 |
| *Corydalis Rhizoma* | MOL004234 | 2,3,9,10-tetramethoxy-13-methyl-5,6-dihydroisoquinolino[2,1-b]isoquinolin-8-one | 76.77 | 0.73 |
| *Corydalis Rhizoma* | MOL001460 | Cryptopin | 78.74 | 0.72 |
| *Corydalis Rhizoma* | MOL004210 | (1S,8'R)-6,7-dimethoxy-2-methylspiro[3,4-dihydroisoquinoline-1,7'-6,8-dihydrocyclopenta[g][1,3]benzodioxole]-8'-ol | 43.95 | 0.72 |
| *Corydalis Rhizoma* | MOL004224 | pontevedrine | 30.28 | 0.71 |
| *Corydalis Rhizoma* | MOL000793 | C09367 | 47.54 | 0.69 |
| *Corydalis Rhizoma* | MOL004191 | Capaurine | 62.91 | 0.69 |
| *Corydalis Rhizoma* | MOL004204 | dehydrocorydaline | 41.98 | 0.68 |
| *Corydalis Rhizoma* | MOL004195 | CORYDALINE | 65.84 | 0.68 |
| *Corydalis Rhizoma* | MOL004214 | isocorybulbine | 40.18 | 0.66 |
| *Corydalis Rhizoma* | MOL000785 | palmatine | 64.60 | 0.65 |
| *Corydalis Rhizoma* | MOL004071 | Hyndarin | 73.94 | 0.64 |
| *Corydalis Rhizoma* | MOL004216 | 13-methylpalmatrubine | 40.97 | 0.63 |
| *Corydalis Rhizoma* | MOL004209 | 13-methyldehydrocorydalmine | 35.94 | 0.63 |
| *Corydalis Rhizoma* | MOL004203 | Dehydrocorybulbine | 46.97 | 0.63 |
| *Corydalis Rhizoma* | MOL004199 | Corynoloxine | 38.12 | 0.60 |
| *Corydalis Rhizoma* | MOL004205 | Dehydrocorydalmine | 43.90 | 0.59 |
| *Corydalis Rhizoma* | MOL000790 | Isocorypalmine | 35.77 | 0.59 |
| *Corydalis Rhizoma* | MOL004196 | Corydalmine | 52.50 | 0.59 |
| *Corydalis Rhizoma* | MOL004221 | norglaucing | 30.35 | 0.56 |
| *Corydalis Rhizoma* | MOL004233 | ST057701 | 31.87 | 0.56 |
| *Corydalis Rhizoma* | MOL004220 | N-methyllaurotetanine | 41.62 | 0.56 |
| *Corydalis Rhizoma* | MOL004197 | Corydine | 37.16 | 0.55 |
| *Corydalis Rhizoma* | MOL000217 | (S)-Scoulerine | 32.28 | 0.54 |
| *Corydalis Rhizoma* | MOL004208 | demethylcorydalmatine | 38.99 | 0.54 |
| *Corydalis Rhizoma* | MOL004193 | Clarkeanidine | 86.65 | 0.54 |
| *Corydalis Rhizoma* | MOL004763 | Izoteolin | 39.53 | 0.51 |
| *Corydalis Rhizoma* | MOL004200 | methyl-[2-(3,4,6,7-tetramethoxy-1-phenanthryl)ethyl]amine | 61.15 | 0.44 |
| *Corydalis Rhizoma* | MOL004232 | tetrahydroprotopapaverine | 57.28 | 0.33 |
| *Corydalis Rhizoma* | MOL000098 | quercetin | 46.43 | 0.28 |
| *Corydalis Rhizoma* | MOL004215 | leonticine | 45.79 | 0.26 |

Supplementary Table 2: 153 common targets.

| Gene | Target name |
| --- | --- |
| XDH | Xanthine dehydrogenase/oxidase |
| ADRA2C | Alpha-2C adrenergic receptor |
| CYCS | Cytochrome c |
| NOS2 | Nitric oxide synthase, inducible |
| SLC6A4 | Sodium-dependent serotonin transporter |
| CHRM5 | Muscarinic acetylcholine receptor M5 |
| GJA1 | Gap junction alpha-1 protein |
| PRKCB | Protein kinase C beta type |
| HTR2C | 5-hydroxytryptamine 2C receptor |
| DPP4 | Dipeptidyl peptidase IV |
| DRD5 | D(1B) dopamine receptor |
| MAOA | Amine oxidase [flavin-containing] A |
| FAS | Tumor necrosis factor receptor superfamily member 6 |
| PON1 | Serum paraoxonase/arylesterase 1 |
| BCL2 | Apoptosis regulator Bcl-2 |
| DRD3 | D(3) dopamine receptor |
| ADRA2A | Alpha-2A adrenergic receptor |
| KCNH2 | Potassium voltage-gated channel subfamily H member 2 |
| CYP1A1 | Cytochrome P450 1A1 |
| HTR3A | 5-hydroxytryptamine receptor 3A |
| APOB | Apolipoprotein B-100 |
| MAPK10 | Mitogen-activated protein kinase 10 |
| CHRM1 | Muscarinic acetylcholine receptor M1 |
| CRP | C-reactive protein |
| GSTP1 | Glutathione S-transferase P |
| CXCL8 | Interleukin-8 |
| SELE | E-selectin |
| ABCB1 | Multidrug resistance protein 1 |
| AHR | Aryl hydrocarbon receptor |
| CHRNA2 | Neuronal acetylcholine receptor subunit alpha-2 |
| NR1I3 | Nuclear receptor subfamily 1 group I member 3 |
| LEPR | Leptin receptor |
| GABBR1 | Gamma-aminobutyric acid type B receptor subunit 1 |
| CHRM4 | Muscarinic acetylcholine receptor M4 |
| CYP19A1 | Cytochrome P450 19A1 |
| MAPK14 | Mitogen-activated protein kinase 14 |
| ADRB1 | Beta-1 adrenergic receptor |
| IL1A | Interleukin-1 alpha |
| GNRH1 | Progonadoliberin-1 |
| DRD2 | D(2) dopamine receptor |
| MPO | Myeloperoxidase |
| SELP | P-selectin |
| ADRA1B | Alpha-1B adrenergic receptor |
| STAT3 | Signal transducer and activator of transcription 3 |
| PTGS2 | Prostaglandin G/H synthase 2 |
| SLPI | Antileukoproteinase |
| CTNNB1 | Catenin beta-1 |
| CCND1 | G1/S-specific cyclin-D1 |
| ESR1 | Estrogen receptor |
| AGTR1 | Type-1 angiotensin II receptor |
| GRM1 | Metabotropic glutamate receptor 1 |
| PDE3A | CGMP-inhibited 3',5'-cyclic phosphodiesterase A |
| ADRB2 | Beta-2 adrenergic receptor |
| POR | NADPH--cytochrome P450 reductase |
| CTSD | Cathepsin D |
| VEGFA | Vascular endothelial growth factor A |
| TGFB1 | Transforming growth factor beta-1 |
| MYC | Myc proto-oncogene protein |
| ADRA1D | Alpha-1D adrenergic receptor |
| VCP | Transitional endoplasmic reticulum ATPase |
| ACHE | Acetylcholinesterase |
| GSK3B | Glycogen synthase kinase-3 beta |
| GPT | Alanine aminotransferase 1 |
| CCL2 | C-C motif chemokine 2 |
| MMP1 | Interstitial collagenase |
| IL6 | Interleukin-6 |
| GABRA1 | Gamma-aminobutyric acid receptor subunit alpha-1 |
| HSP90AA1 | Heat shock protein HSP 90 |
| CASP3 | Caspase-3 |
| PARP1 | Poly [ADP-ribose] polymerase 1 |
| CD14 | Monocyte differentiation antigen CD14 |
| HMGCR | 3-hydroxy-3-methylglutaryl-coenzyme A reductase |
| NR3C2 | Mineralocorticoid receptor |
| GSR | Glutathione reductase, mitochondrial |
| PGP | Phosphoglycolate phosphatase |
| SULT1E1 | Estrogen sulfotransferase |
| HMOX1 | Heme oxygenase 1 |
| INS | Insulin |
| MAOB | Amine oxidase [flavin-containing] B |
| CYP1A2 | Cytochrome P450 1A2 |
| CRH | Corticoliberin |
| IL10 | Interleukin-10 |
| MAPK1 | Mitogen-activated protein kinase 1 |
| HTR1B | 5-hydroxytryptamine 1B receptor |
| ADRA2B | Alpha-2B adrenergic receptor |
| PLAT | Tissue-type plasminogen activator |
| SOD1 | Superoxide dismutase [Cu-Zn] |
| IL2 | Interleukin-2 |
| ERBB3 | Receptor tyrosine-protein kinase erbB-3 |
| ALDH3A1 | Aldehyde dehydrogenase, dimeric NADP-preferring |
| GRIN2D | Glutamate [NMDA] receptor subunit epsilon-4 |
| FOS | Proto-oncogene c-Fos |
| IFNG | Interferon gamma |
| HSPA5 | 78 kDa glucose-regulated protein |
| IL4 | Interleukin-4 |
| GRM5 | Metabotropic glutamate receptor 5 |
| OPRD1 | Delta-type opioid receptor |
| MAPK8 | Mitogen-activated protein kinase 8 |
| ICAM1 | Intercellular adhesion molecule 1 |
| ABAT | 4-aminobutyrate aminotransferase, mitochondrial |
| F2R | Proteinase-activated receptor 1 |
| MAPK3 | Mitogen-activated protein kinase 3 |
| AKR1B1 | Aldose reductase |
| NCOA1 | Nuclear receptor coactivator 1 |
| DRD4 | D(4) dopamine receptor |
| HIF1A | Hypoxia-inducible factor 1-alpha |
| SLC2A4 | Solute carrier family 2, facilitated glucose transporter member 4 |
| MAP2 | Microtubule-associated protein 2 |
| PDE10A | cAMP and cAMP-inhibited cGMP 3',5'-cyclic phosphodiesterase 10A |
| CHRM2 | Muscarinic acetylcholine receptor M2 |
| ODC1 | Ornithine decarboxylase |
| OPRM1 | Mu-type opioid receptor |
| TYR | Tyrosinase |
| SERPINE1 | Plasminogen activator inhibitor 1 |
| VCAM1 | Vascular cell adhesion protein 1 |
| TP53 | Cellular tumor antigen p53 |
| IFNA1 | Interferon alpha-1/13 |
| CASP9 | Caspase-9 |
| CDKN1A | Cyclin-dependent kinase inhibitor 1 |
| CAT | Catalase |
| DIO1 | Type I iodothyronine deiodinase |
| RASA1 | Ras GTPase-activating protein 1 |
| NCOA2 | Nuclear receptor coactivator 2 |
| AKT1 | RAC-alpha serine/threonine-protein kinase |
| ADCY2 | Adenylate cyclase type 2 |
| CYP2B6 | Cytochrome P450 2B6 |
| SIRT1 | NAD-dependent deacetylase sirtuin-1 |
| ABCC1 | Multidrug resistance-associated protein 1 |
| IL1B | Interleukin-1 beta |
| CHRM3 | Muscarinic acetylcholine receptor M3 |
| AKR1C3 | Aldo-keto reductase family 1 member C3 |
| NFKBIA | NF-kappa-B inhibitor alpha |
| IGFBP3 | Insulin-like growth factor-binding protein 3 |
| ALB | Serum albumin |
| F3 | Tissue factor |
| HTR2A | 5-hydroxytryptamine 2A receptor |
| NR1I2 | Nuclear receptor subfamily 1 group I member 2 |
| IFNB1 | Interferon beta |
| JUN | Transcription factor AP-1 |
| MMP10 | Stromelysin-2 |
| ESR2 | Estrogen receptor beta |
| BAX | Apoptosis regulator BAX |
| CD40LG | CD40 ligand |
| CYP3A4 | Cytochrome P450 3A4 |
| SCN5A | Sodium channel protein type 5 subunit alpha |
| APP | Amyloid beta A4 protein |
| GRIA2 | Glutamate receptor 2 |
| SLC6A3 | Sodium-dependent dopamine transporter |
| DRD1 | Dopamine D1 receptor |
| ADIPOQ | Adiponectin |
| SLC6A2 | Sodium-dependent noradrenaline transporter |
| ADH1B | Alcohol dehydrogenase IB |
| ADH1C | Alcohol dehydrogenase IC |

# Supplementary Figures


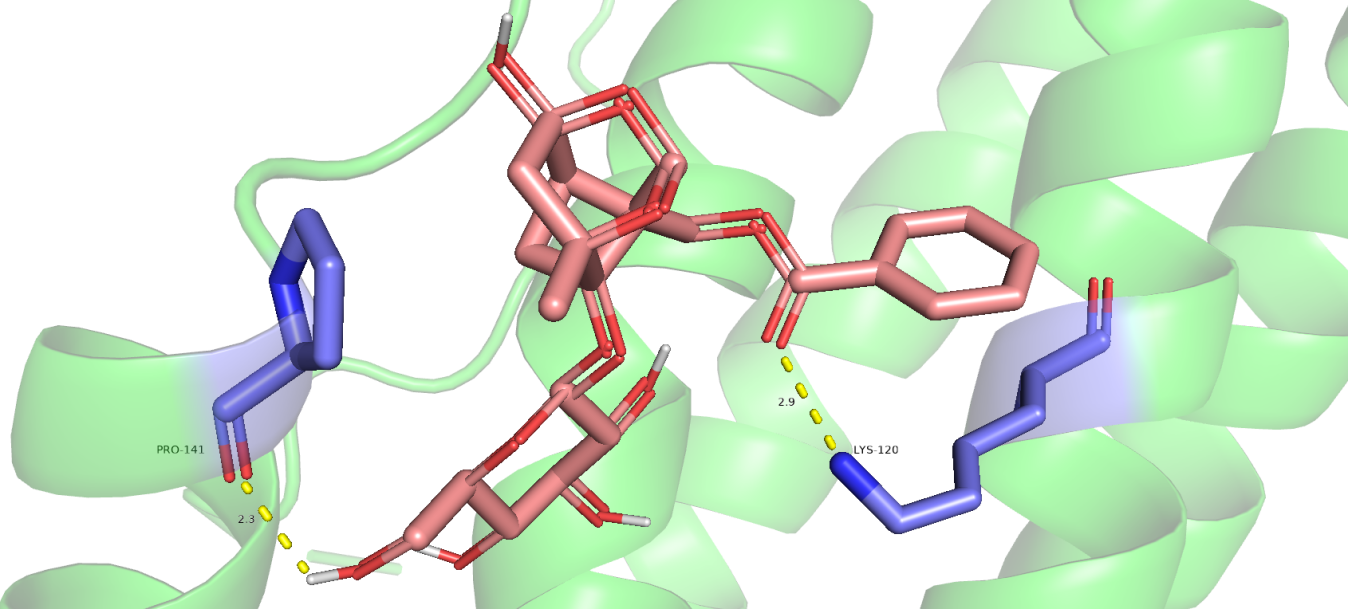


(A)IL6-quercetin


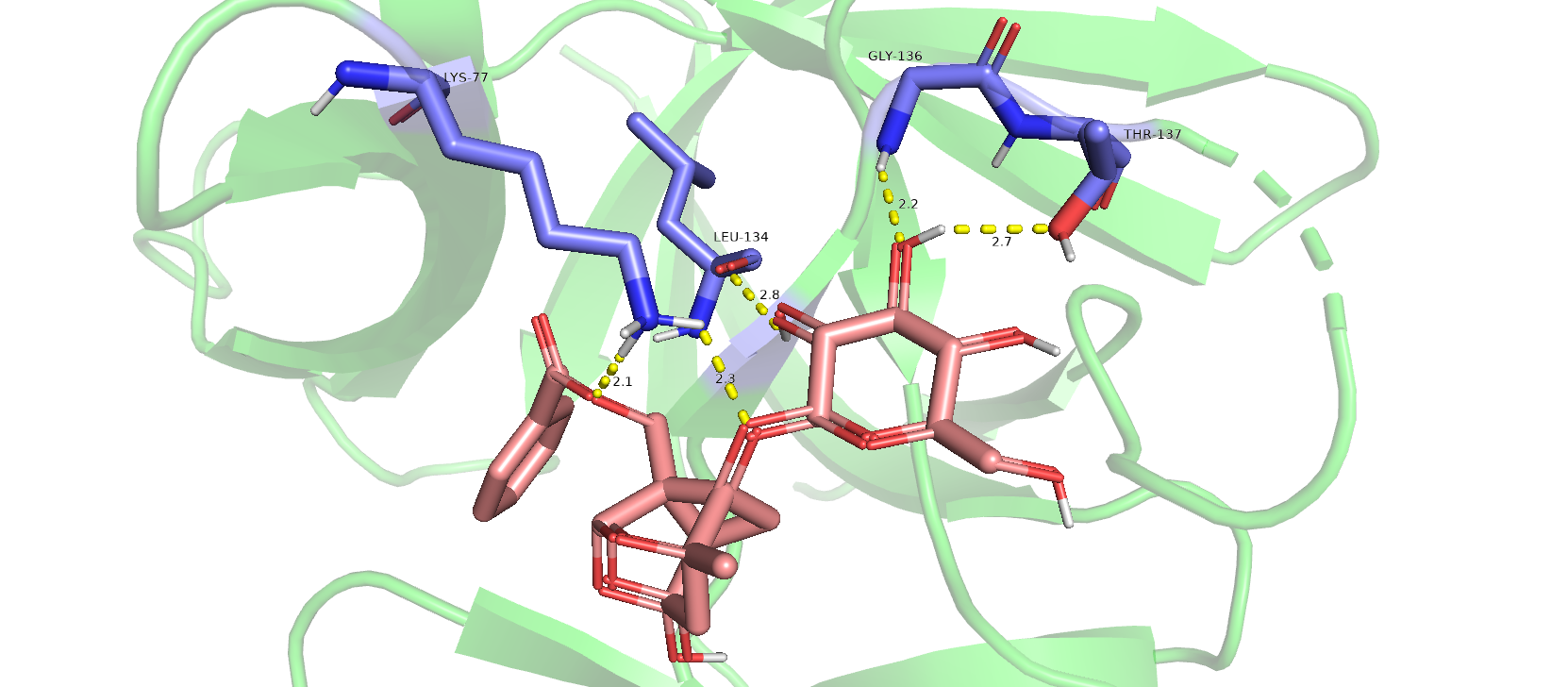


(B)IL1B-quercetin


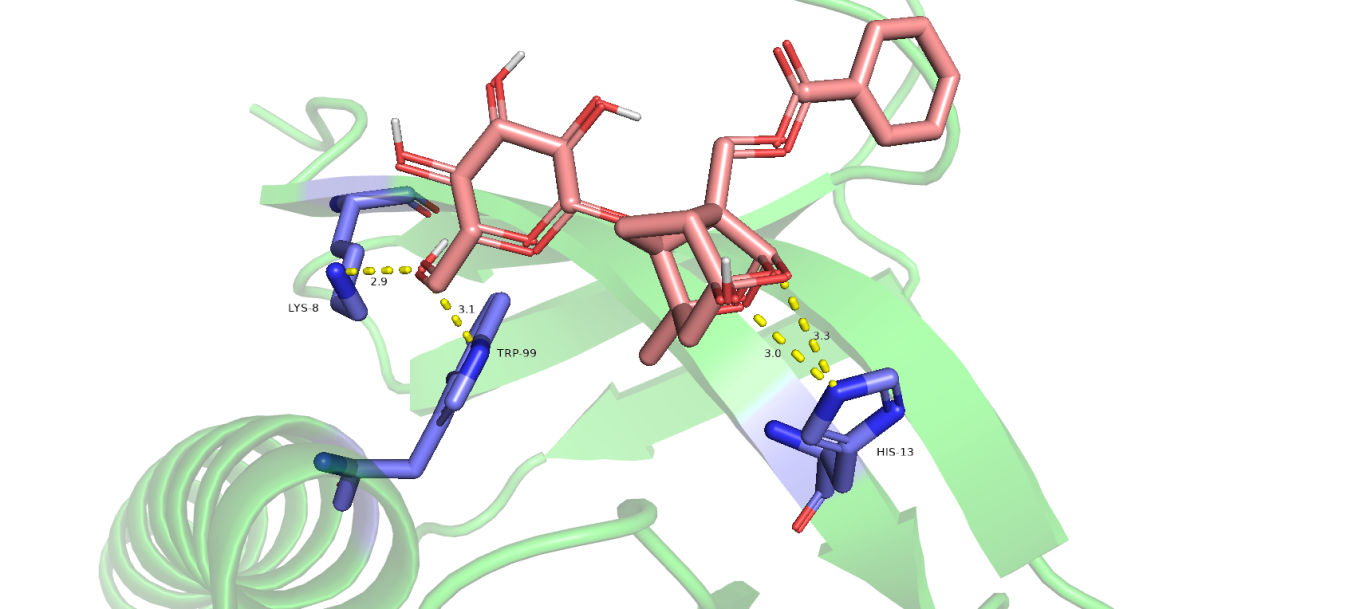


(C)AKT1-quercetin


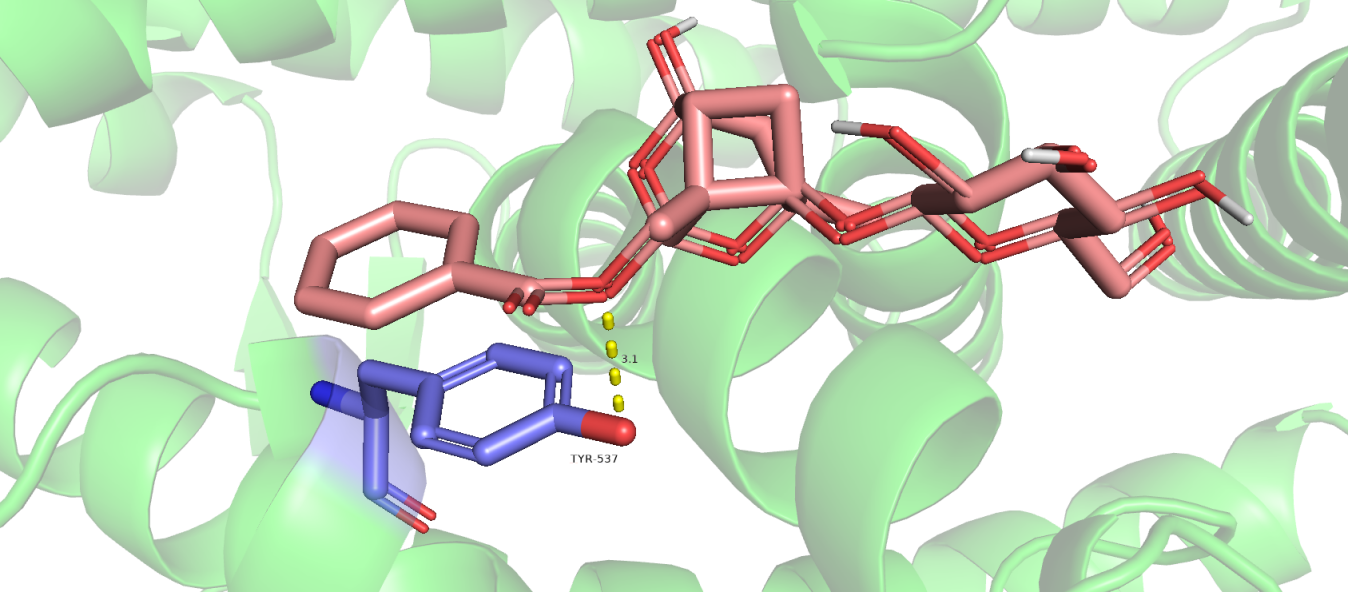


(D)TP53-quercetin


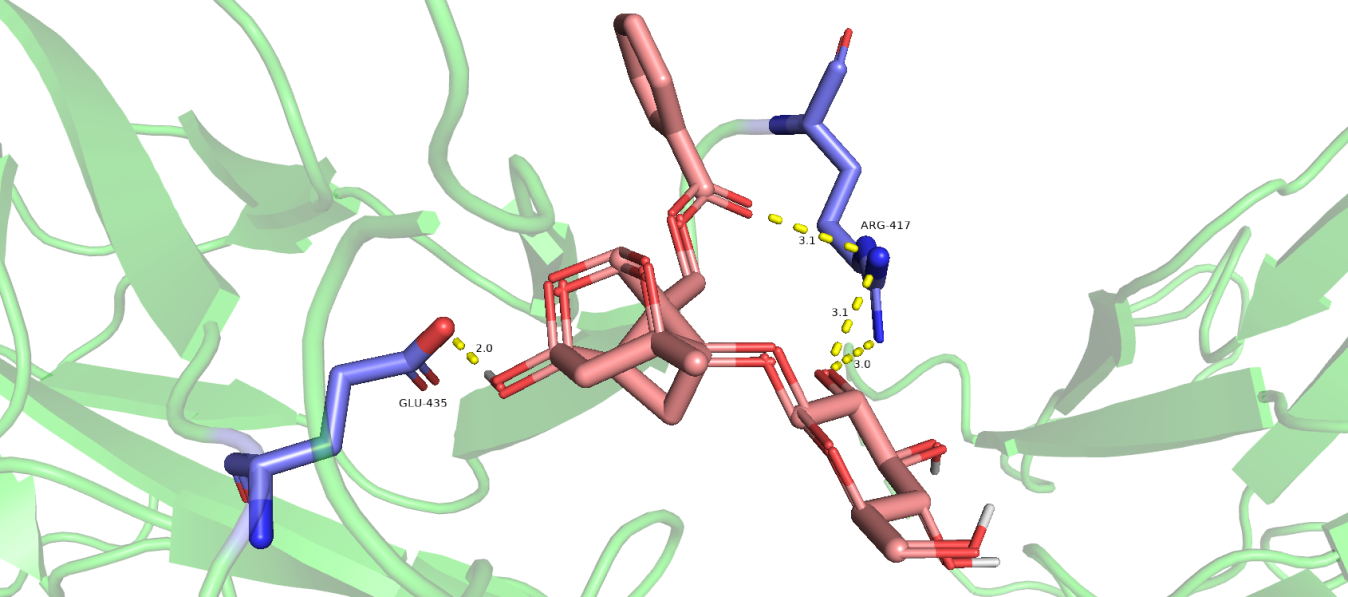


(E)STAT3-quercetin


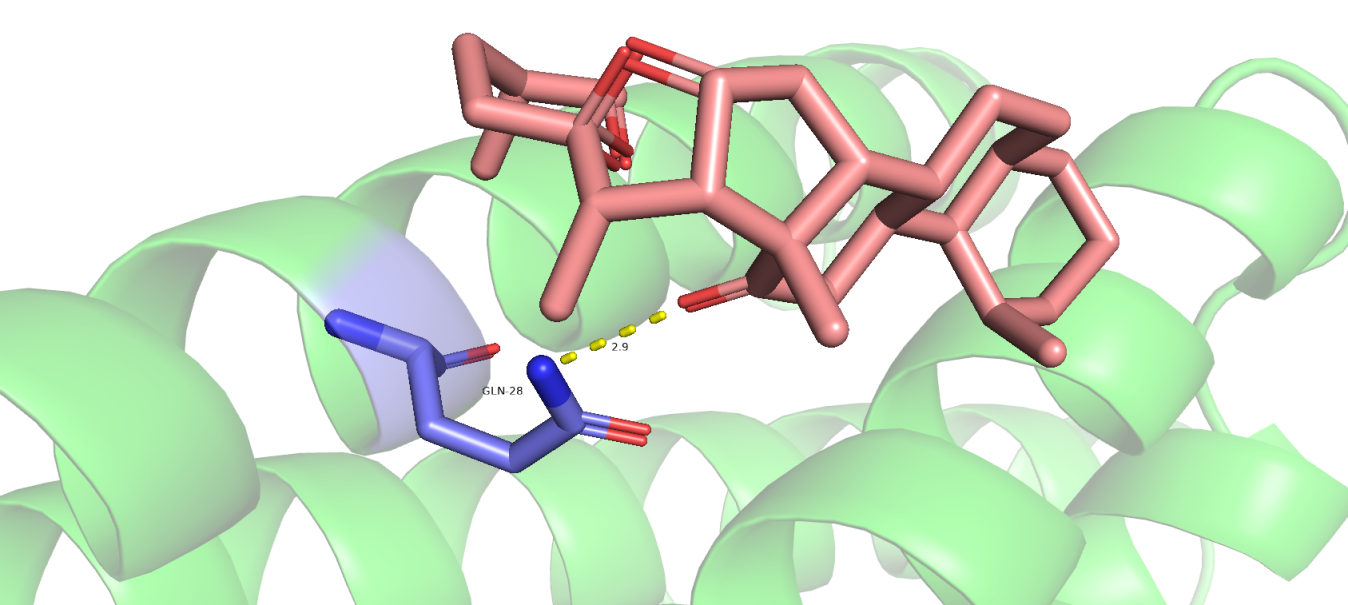


(F)IL6-beta sitosterol


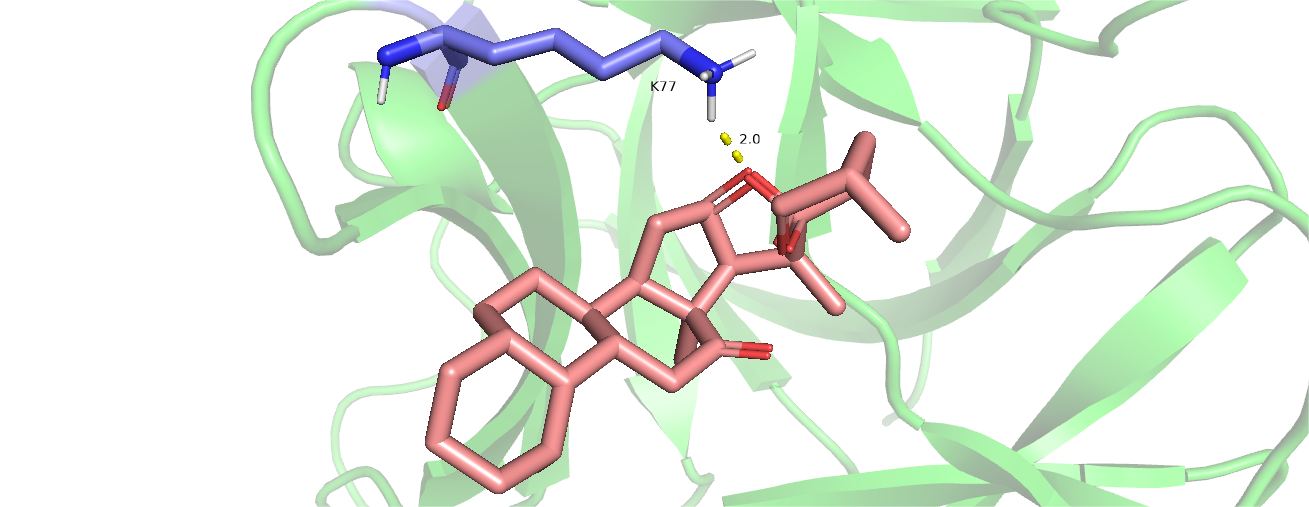


(G)IL1B-beta sitosterol


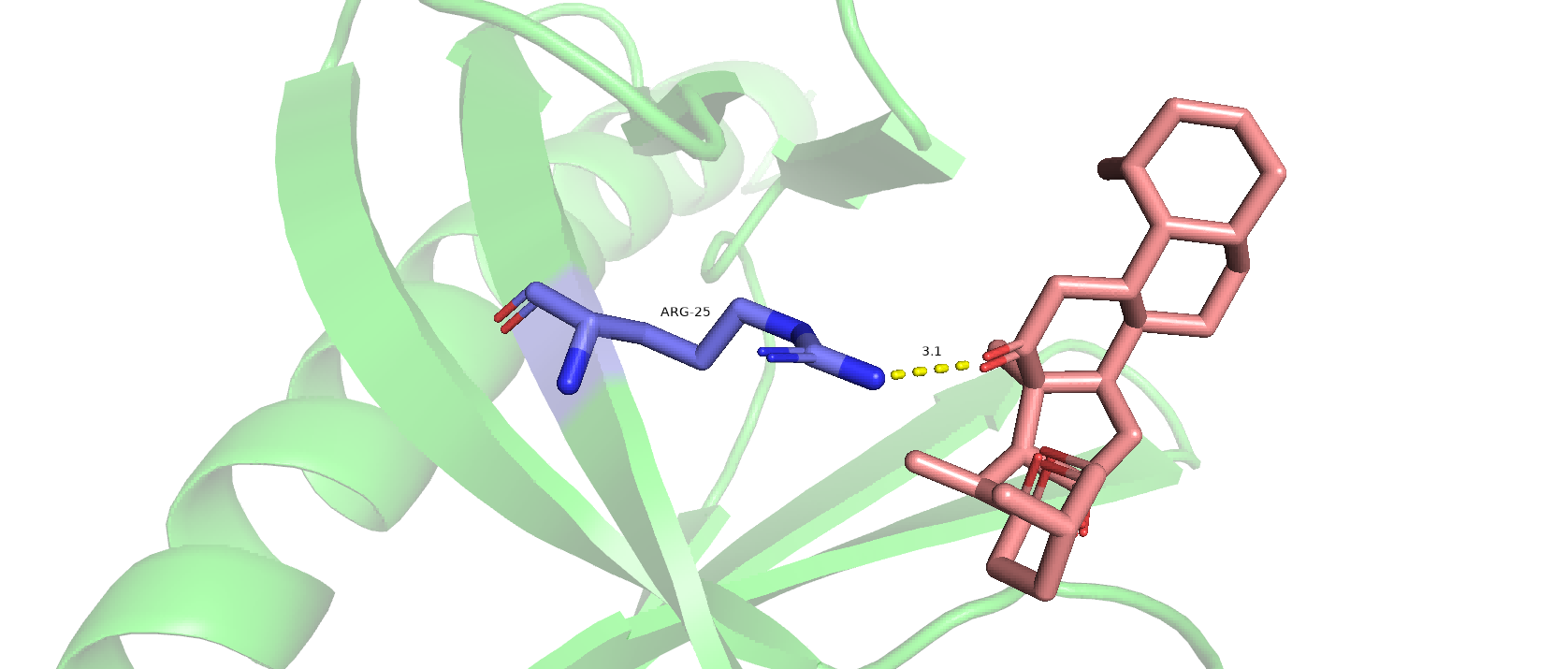


(H)AKT1-beta sitosterol


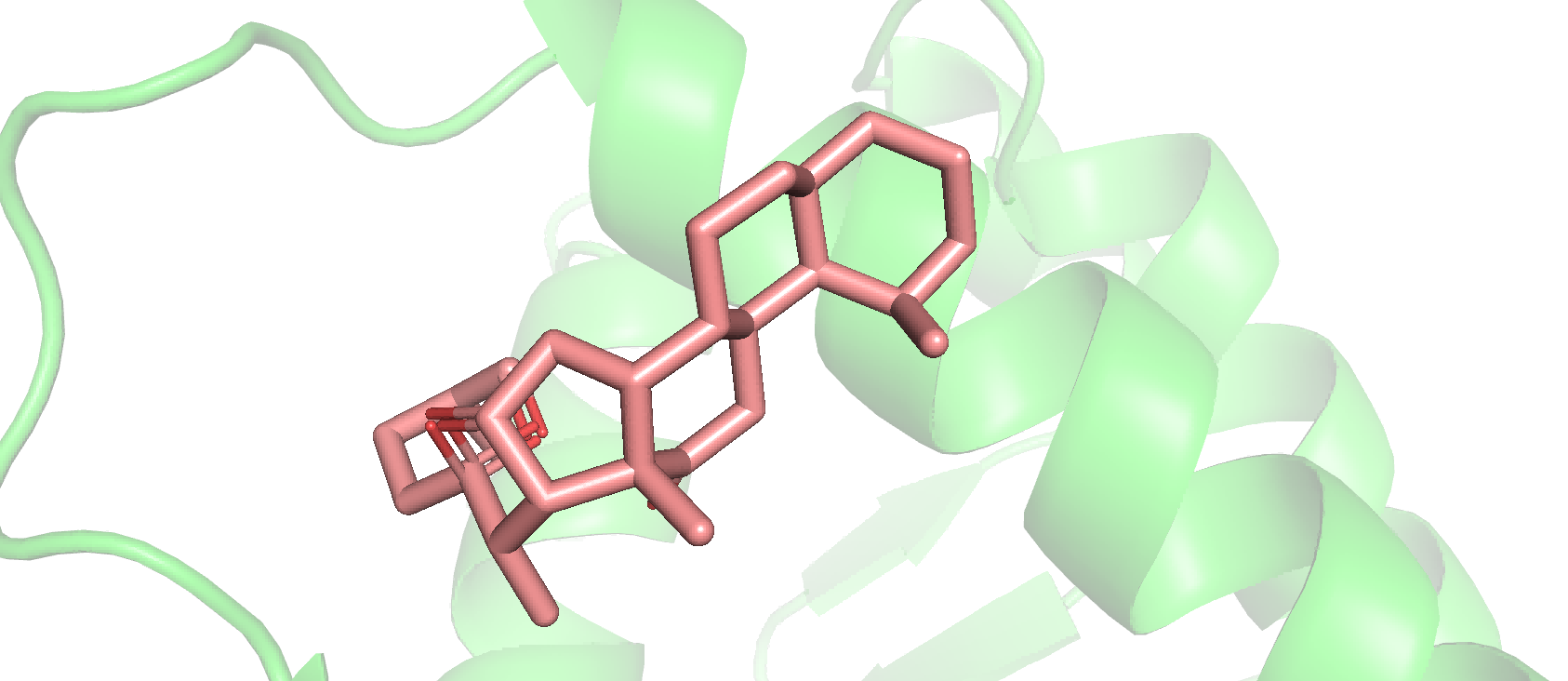


(I)TP53-beta sitosterol


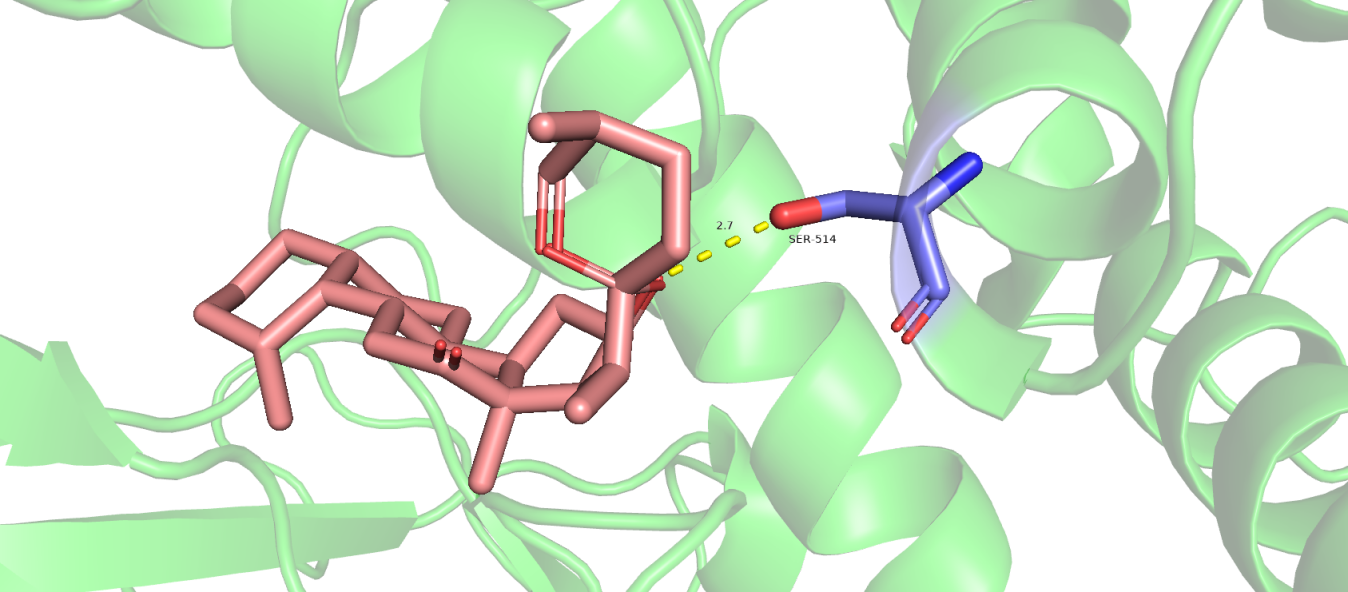


(J)STAT3-beta sitosterol


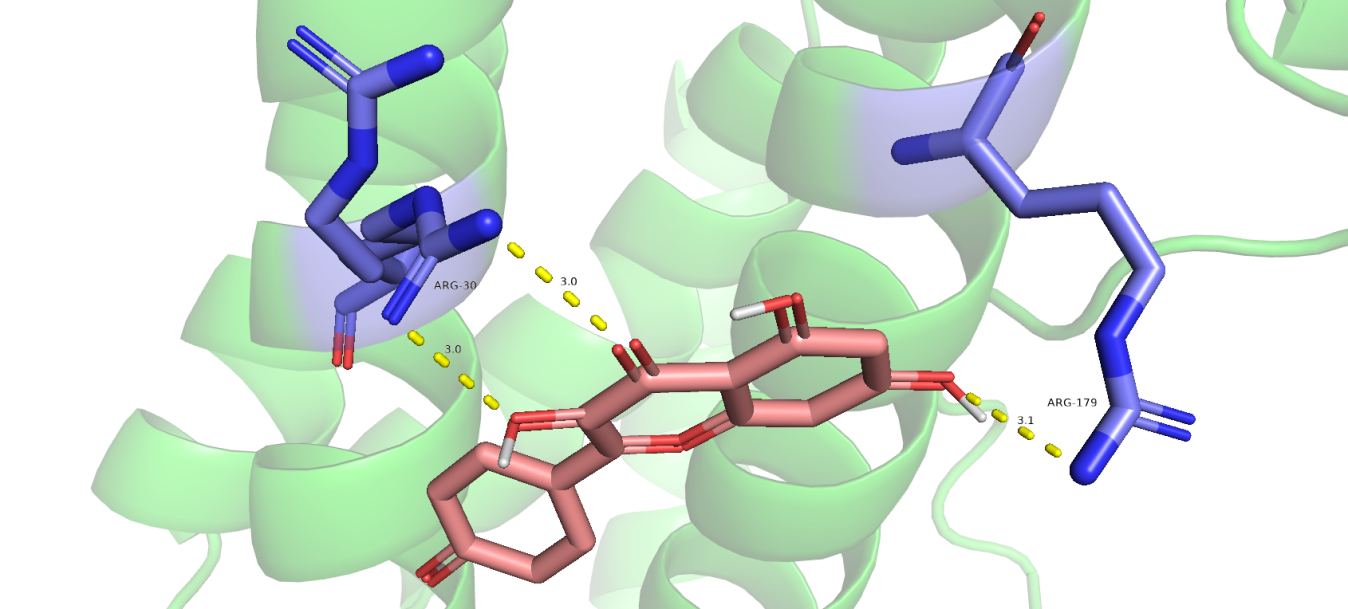


(K)IL6-kaempferol


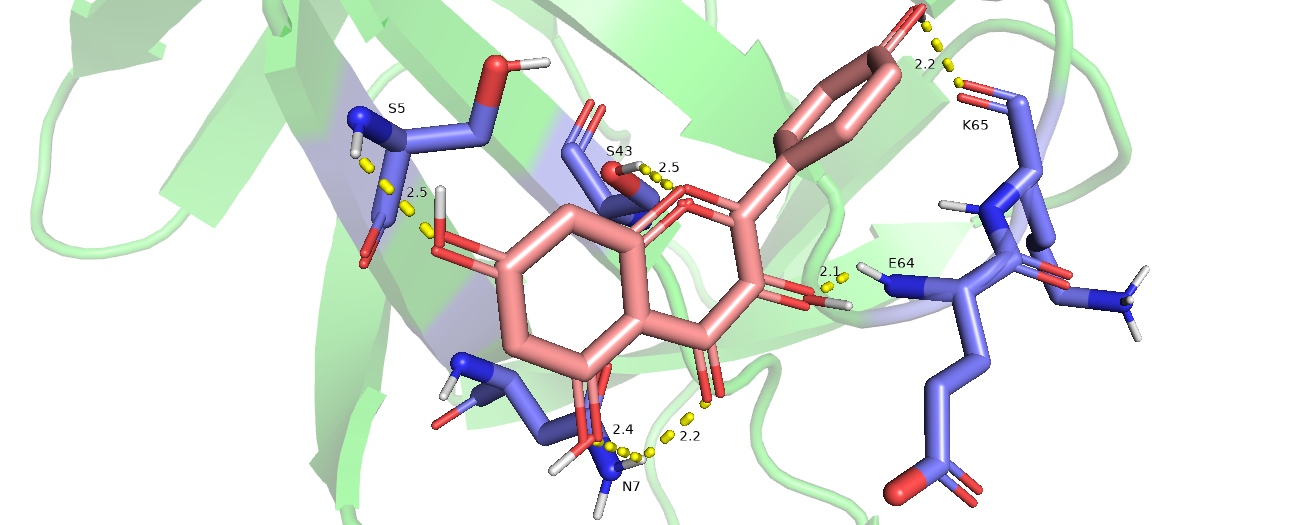


(L)IL1B-kaempferol


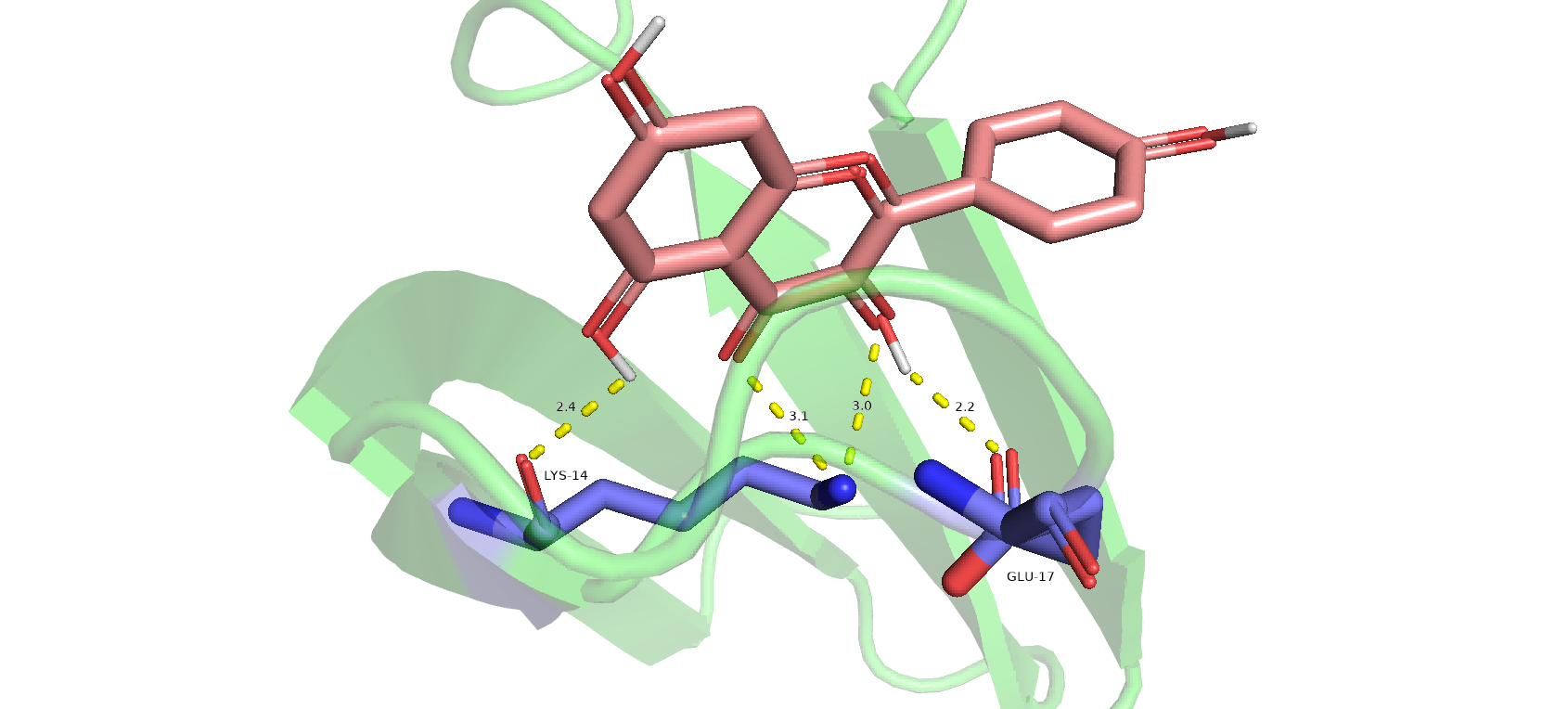


(M)AKT1-kaempferol


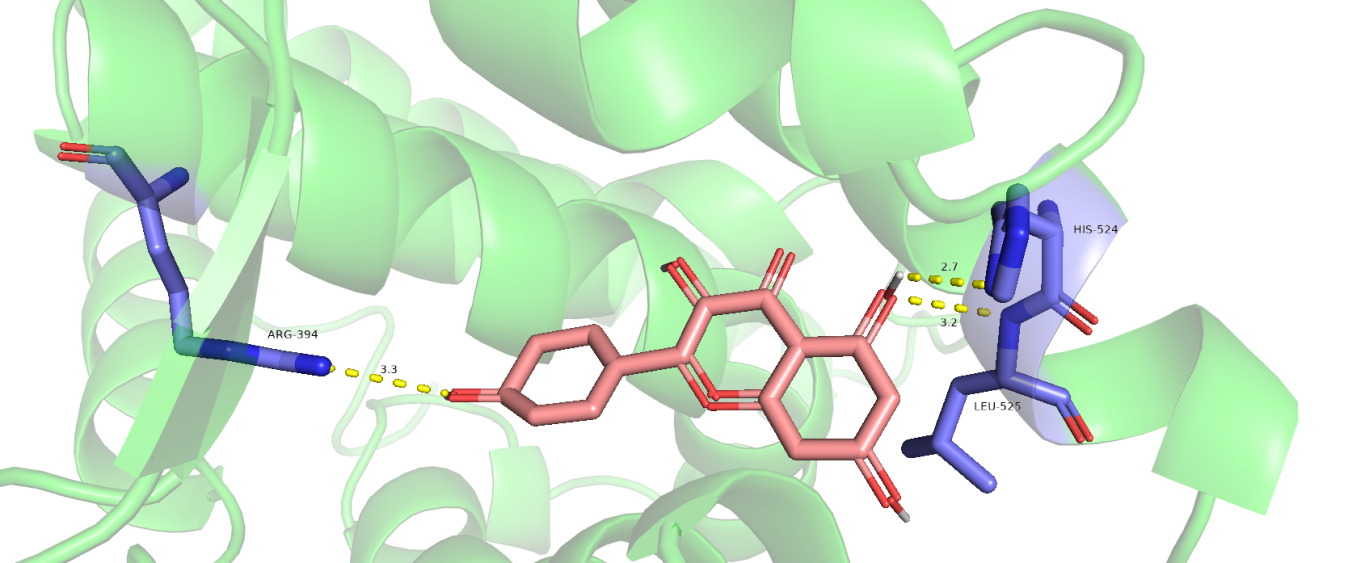


(N)TP53-kaempferol


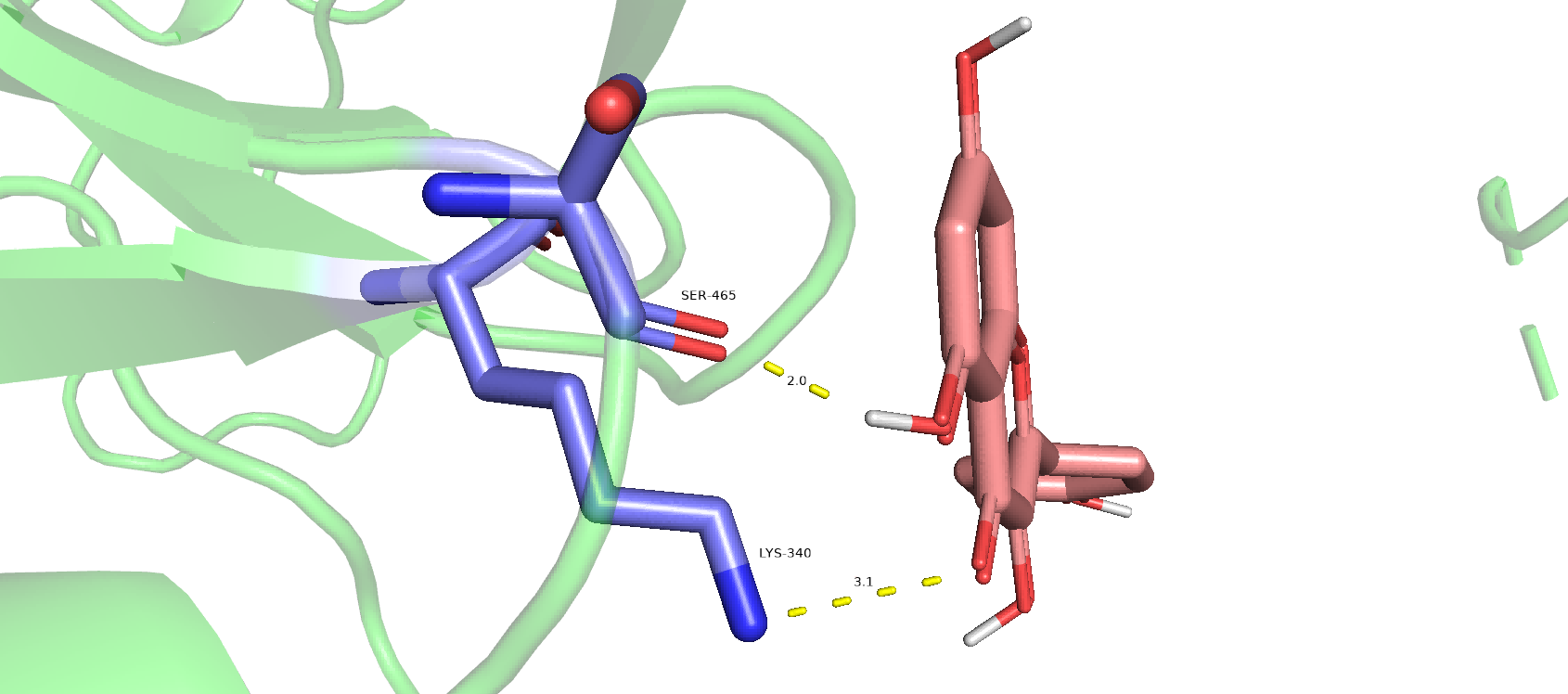


(O)STAT3-kaempferol

**Supplementary Figure 1.** The figure of molecular docking between active substances and proteins. (A)IL6-quercetin; (B)IL1B-quercetin; (C)AKT1-quercetin; (D)TP53-quercetin; (E)STAT3-quercetin; (F)IL6-beta sitosterol; (G)IL1B-beta sitosterol; (H)AKT1-beta sitosterol; (I)TP53-beta sitosterol; (J)STAT3-beta sitosterol; (K)IL6-kaempferol; (L)IL1B-kaempferol; (M)AKT1-kaempferol; (N)TP53-kaempferol; (O)STAT3-kaempferol.
